# Plant Nitrate Reductases Regulate Nitric Oxide Production and Nitrogen-Fixing Metabolism During the *Medicago truncatula–Sinorhizobium meliloti* Symbiosis

**DOI:** 10.3389/fpls.2020.01313

**Published:** 2020-09-04

**Authors:** Antoine Berger, Alexandre Boscari, Natasha Horta Araújo, Mickaël Maucourt, Mohamed Hanchi, Stéphane Bernillon, Dominique Rolin, Alain Puppo, Renaud Brouquisse

**Affiliations:** ^1^Institut Sophia Agrobiotech, UMR INRAE 1355, Université Côte d’Azur, CNRS, Sophia Antipolis, France; ^2^Department of Horticultural Science, University of Florida, Gainesville, FL, United States; ^3^Univ. Bordeaux INRAE, UMR Biologie du Fruit et Pathologie, Villenave d’Ornon, France; ^4^PMB-Metabolome, INRAE, Bordeaux Metabolome Facility, Villenave d’Ornon, France

**Keywords:** hypoxia, legumes, *Medicago truncatula*, nitric oxide, nitrogen-fixing symbiosis, nitrate reductase, nodules

## Abstract

Nitrate reductase (NR) is the first enzyme of the nitrogen reduction pathway in plants, leading to the production of ammonia. However, in the nitrogen-fixing symbiosis between legumes and rhizobia, atmospheric nitrogen (N_2_) is directly reduced to ammonia by the bacterial nitrogenase, which questions the role of NR in symbiosis. Next to that, NR is the best-characterized source of nitric oxide (NO) in plants, and NO is known to be produced during the symbiosis. In the present study, we first surveyed the three NR genes (Mt*NR1*, Mt*NR2*, and Mt*NR3*) present in the *Medicago truncatula* genome and addressed their expression, activity, and potential involvement in NO production during the symbiosis between *M. truncatula* and *Sinorhizobium meliloti*. Our results show that Mt*NR1* and Mt*NR2* gene expression and activity are correlated with NO production throughout the symbiotic process and that MtNR1 is particularly involved in NO production in mature nodules. Moreover, NRs are involved together with the mitochondrial electron transfer chain in NO production throughout the symbiotic process and energy regeneration in N_2_-fixing nodules. Using an *in vivo* NMR spectrometric approach, we show that, in mature nodules, NRs participate also in the regulation of energy state, cytosolic pH, carbon and nitrogen metabolism under both normoxia and hypoxia. These data point to the importance of NR activity for the N_2_-fixing symbiosis and provide a first explanation of its role in this process.

## Introduction

In plants, yeasts, algae, and fungi, nitrate reductase (NR) is a key enzyme of the nitrogen (N) reduction and assimilation pathway. It catalyzes the reduction of nitrate (NO_3_^−^) to nitrite (NO_2_^−^), which is itself reduced to ammonia (NH_4_^+^) by nitrite reductase (NiR), before being assimilated into the amino acids and the nitrogen compounds of the cell (Campbell, 1999). Sixty-five million years ago, the legume family developed a beneficial mutual relationship with soil bacteria, the Rhizobia, which directly reduce atmospheric nitrogen (N_2_) to ammonia (NH_4_^+^) through the activity of the nitrogenase under nitrogen deficiency situations (Wang et al., 2018). In exchange for NH_4_^+^, plants supply “board and lodging” for the bacteria, providing them with an ecological niche for their development and carbon nutrients for their functioning, in neoformed organs called nodules (Udvardi and Day, 1997; Terpolilli et al., 2012). Thus, in legume nodules, the bacterial nitrogenase substitutes for the NR–NiR pathway to produce NH_4_^+^ and supply the plant with reduced nitrogen. However, many studies have reported high NR expression and activity in symbiotic nodules (see Streeter, 1985a; Streeter, 1985b; Arrese-Igor et al., 1990; Silveira et al., 2001; Kato et al., 2003; Sanchez et al., 2010; Horchani et al., 2011, and references therein) and the question arose—and still arises—of what the NR can be used in the N_2_-fixing symbiosis, where nitrogen reduction is ensured by nitrogenase.

The involvement of NR activity in NO production has been evidenced in many plant organs and tissues over the past 20 years (Dean and Harper, 1988; Yamasaki and Sakihama, 2000; Rockel et al., 2002; Sakihama et al., 2002; Gupta et al., 2005; Planchet et al., 2005; Kato et al., 2010; Kolbert et al., 2010). Until recently, NR was considered to produce directly NO *via* the reduction of NO_2_^−^, but another indirect mechanism of NO synthesis involving NR has been proposed (Chamizo-Ampudia et al., 2016; Chamizo-Ampudia et al., 2017). In this mechanism, through its diaphorase activity, NR transfers electron from NAD(P)H to a NO-forming nitrite reductase (NOFNiR) that catalyzes the reduction of NO_2_^−^ to NO. Although the interaction between NR and NOFNiR has been argued in eukaryotic algae, the proof of concept in higher plant is still not demonstrated. NO is a reactive free radical gaseous molecule with a broad spectrum of regulatory functions in plant growth and development, and in response to abiotic and biotic factors (Kolbert et al., 2019). NO is particularly involved in the legume–Rhizobium symbiotic interactions (Hichri et al., 2015; Berger et al., 2019).

During the first hours after inoculation with the symbiotic partner, NO was observed in the roots of *Lotus japonicus*, *Medicago sativa*, and *Medicago truncatula* (Nagata et al., 2008; Fukudome et al., 2016; Hichri et al., 2016). Its production was also detected during the infection process along the infection thread and in the dividing cells of the *M. truncatula* nodule primordium (del Giudice et al., 2011). Similar results were observed in other Medicago species (Pii et al., 2007). In *M. truncatula* mature nodules, NO has been shown to accumulate particularly in the N_2_-fixing zone (Baudouin et al., 2006; Hichri et al., 2016), and at the onset of nodule senescence a NO production was reported at the junction of the N_2_-fixing and senescence zones (Cam et al., 2012). A recent study with *M. truncatula* showed that NO is produced throughout the whole symbiotic process, from infection with *Sinorhizobium meliloti* up to, at least, 8 weeks post-inoculation (wpi), exhibiting production peaks during the first hours of the symbiotic interaction, during early development of the nodule and when the nodule becomes mature (Berger et al., 2020). These observations suggest that NO performs specific signaling and/or metabolic functions during symbiosis. Indeed, two transcriptomic analyses led to the identification of NO-responsive genes either in 4 days post-inoculation (dpi) roots (Boscari et al., 2013) or in developing and mature nodules (Ferrarini et al., 2008). More than 400 plant genes are NO-regulated during the symbiotic process, including genes involved in nodule development normally induced by the symbiont, suggesting that NO participate in signal transduction in the plant–microorganism interaction (Ferrarini et al., 2008; Boscari et al., 2013). On the bacterial partner side, NO has also been shown to regulate a hundred genes, most of them being similarly regulated under microoxic conditions (Bobik et al., 2006; Meilhoc et al., 2010). The biological activity of NO is particularly mediated through redox-dependent protein modifications such as S-nitrosation, tyrosine nitration and metal nitrosylation (Besson-Bard et al., 2008; Hancock, 2019). Several key proteins involved in nodule primary metabolism or stress response were reported to be S-nitrosated, indicating a crucial role of NO in the energy, carbon, and nitrogen metabolism (Puppo et al., 2013). Among these proteins, enzymes such as glutathione peroxidase (Castella et al., 2017), glutamine synthetase (Melo et al., 2011; Seabra and Carvalho, 2015), and leghemoglobins (Mathieu et al., 1998; Navascues et al., 2012; Sainz et al., 2015; Becana et al., 2020) have been shown to be differently regulated by various NO-dependent modifications. Finally, NO has also been shown to play a metabolic function in the maintenance of energy status under hypoxic conditions, such as that prevailing in microoxic nodules (Igamberdiev and Hill, 2004; Igamberdiev and Hill, 2009). Indeed, NO is involved in a respiratory cycle, called Phytoglobin-NO respiration (PNR), allowing the regeneration of ATP under low oxygen concentrations. PNR is divided into four steps including: 1) NO_3_^−^ reduction to NO_2_^−^ by cytosolic NR, 2) NO_2_^−^ transport from the cytosol to the mitochondrial matrix, 3) NO_2_^−^ reduction to NO by the mitochondrial electron transfer chain, and 4) NO diffusion to the cytosol and oxidation to NO_3_^−^ by Phytoglobins. Evidence indicates that PNR potentially functions and participates in the regeneration of ATP in N_2_-fixing nodules (Horchani et al., 2011; Berger et al., 2020). Thus, the importance and diversity of NO functions in symbiosis, whether through the regulation of gene expression, the modulation of enzyme activity, or its involvement in energy metabolism, require that its production should be timely and tightly regulated during the symbiotic process. Considering that the NR pathway is the main NO production pathway in plants and that its activity is high in nodules, we hypothesized that NR is significantly involved in the production of NO during the symbiotic process.

The aim of this work was to test this hypothesis. To this end, we addressed the expression and activity of the three NR genes present in the *M. truncatula* genome, from the first hours of symbiotic interaction up to 8 wpi, at the onset of nodule senescence. Then, we investigated the impact of NR activity on NO production and energy regeneration during nodule development. Last, we studied its role in carbon and nitrogen metabolism in mature nodules. Based on our data, we discuss the potential roles of NR on NO homeostasis during the symbiotic process and more generally on the N_2_-fixing process.

## Materials and Methods

### Plants Growth and Inoculation Conditions

*Medicago truncatula* (cv Jemalong A17) were scarified, sterilized, and germinated as in del Giudice et al. (2011). Seedlings were cultivated and inoculated with *Sinorhizobium meliloti* 2011 strain either in Petri dishes as in del Giudice et al. (2011), or in planters as in Horchani et al. (2011). A basic intake of 0.2 mM KNO_3_ is provided to plants on Petri dishes and planters. Cultures in Petri dishes were used for short-term experiments up to 14 days post-inoculation (dpi), while those in planters were used for long-term experiments up to 8 weeks post-inoculation (wpi). Roots and/or nodules were harvested at various times of the kinetics. For short-term experiments, 2 cm-long root segments corresponding to the infection zone (del Giudice et al., 2011) were harvested for gene expression and NO production. For long-term experiments only nodules were used.

### NR Sequences Acquisition and Analyses

Protein sequences of NR were obtained from three genomic and protein databases: NCBI (www.blast.ncbi.nlm.nih.gov), Phytozome (www.phytozome.jgi.doe.gov), and Uniprot (www.uniprot.org). Local or multiple alignment search tools (BLAST from NCBI and Phytozome; Water and ClustalW from the European Bioinformatic Institute platform EBI) were used to analyze the sequence’s quality, length, and uniqueness. Sequences kept were listed in **Supplementary Table S1**, where a nomenclature code was assigned to each protein in order to simplify data-reading in the phylogenetic tree. The code is made of the name of the species represented, the name allotted to the sequence on the literature or NCBI, and the number of amino acids composing each protein.

### Construction of the Phylogenetic Tree

NR sequences were aligned using Muscle algorithm (Madeira et al., 2019), and their evolutionary history was inferred by Maximum Likelihood (ML) and the JTT matrix-based model (Jones et al., 1992) using MegaX software (Kumar et al., 2018). Initial trees calculated for the heuristic search were acquired automatically by applying Neighbor-Join (NJ) and BioNJ algorithms to a pair-wise distance matrix approximated using the JTT model. The number of distinct/identical residues between each pair of sequences in the multiple alignment was therefore calculated to construct the matrix, and the topology with a higher log likelihood value was selected. The tree was inferred by Nearest-Neighbor-Interchange (NIN) heuristic method, and to assess the reliability of the inference, the bootstrapping method was applied. The consensus tree deduced from 1,000 bootstrap repetitions was retained, and branches corresponding to divisions reproduced in less than 50% of the bootstrap repetitions were not considered. An outgroup, *Chlamydomonas reinhardtii* ‘s NR NIT1, was used to root the tree.

### Construction of a Binary Vector for Hairy Root Transformation

For promoter transcriptional fusions, fragments of 1,647, 1,700, and 1,554 bp upstream of the start codon were amplified by PCR using the primers indicated in **Table S2** for Mt*NR1*, Mt*NR2*, and Mt*NR3* respectively. Each PCR fragment was first cloned into the pDONR207 donor vector and then into the plant expression vector pKGWFS7 (Karimi et al., 2002) using Gateway technology (Invitrogen, http://www.invitrogen.com). For the RNA interference (RNAi) construct, 432- and 441-bp fragments of Mt*NR1* (MtrunA17Chr3g0115151) and Mt*NR2* (MtrunA17Chr5g0424491) genes were amplified *via* polymerase chain reaction (PCR) with specific primers (**Table S2**). PCR products were independently ligated into pGEM-T easy vector (Promega) and subsequently subcloned into pENTR4 vectors in *Bam*HI–*Kpn*I restriction sites for Mt*NR1* and *Eco*RI and *Kpn*I restriction sites for Mt*NR2*. The pENTR4 vector carrying the Mt*NR1* or the Mt*NR2* fragment was recombined with pK7GWIWG5D(II) vector (Horchani et al., 2011) using the LR clonase enzyme mix (catalog no. 11791-019; Invitrogen) to create the RNAi expression vectors. As control, transgenic roots transformed with empty pK7GWIWG5D(II) vector and selected on the base of green fluorescent protein (GFP) marker expression were used. Constructs were checked by sequencing, introduced by electroporation into *Agrobacterium rhizogenes* strain ARqua1, and used for *M. truncatula* root transformation as described by Boisson-Dernier et al. (2001).

### Measurement of NO Production

NO detection was performed as in Horchani et al. (2011) using the 4,5-diaminofluorescein probe (DAF-2, Sigma-Aldrich) with the following changes. Either nodules (20–30 mg fresh weight) or root segments (50–100 mg fresh weight) were incubated in the dark at 23°C in 1 ml of detection buffer (10 mM Tris-HCl pH 7.4, 10 mM KCl) in the presence of 10 μM DAF-2. As a control, NO production was measured in the same experimental system through the use of the Cu(II) fluorescein (CuFL) fluorescent probe (Strem Chemicals) instead of DAF-2 in the detection buffer as described in Horchani et al. (2011). Similar results were obtained with both probes. The production of NO was measured with a spectrofluorimeter-luminometer (Xenius, SAFAS, Monaco). Inhibitors are used as described in Horchani et al. (2011). The inhibitors are added to the reaction medium for the determination of NO at the concentration of 1 mM tungstate (Tg), 1 mM allopurinol, 1 mM propyl gallate, and 300 μM potassium cyanide (KCN). NO production was initiated 1 h after the addition of inhibitors. Three independent biological replicates have been performed with three technical replicates per biological assay.

### Nitrogen-Fixing Capacity Measurement

Nitrogenase activity of the nodules was determined *in vivo* by measuring acetylene reducing activity (ARA, Hardy et al., 1968). Nodulated roots were harvested and incubated at 30°C for 1 h in rubber-capped tubes containing 10% acetylene atmosphere. Ethylene concentrations were determined by gas chromatography (Agilent GC 6890N, Agilent Technologies) equipped with a GS-Alumina separating capillary column. Three independent biological replicates have been performed with five technical replicates per biological assay.

### Phosphorus NMR

For each experiment, 0.9 to 1.1 g fresh weight of 4 wpi-old nodules (around 1,400 to 1,700 nodules) were harvested and incubated at ambient temperature in an aerated perfusion medium containing 1 mM KH_2_PO_4_, 1 mM MgSO_4_,7H_2_O, 0.25 mM K_2_SO_4_, 0.5 mM CaCl_2_, 10 mM MES/KOH, pH 6.0, and 25 mM glucose. At the end of the preparation period of approximately 3 h, the nodules were placed between two filters into a 10-mm tightly closed NMR tube, part of a homebuilt perfusion system. The latter, evolved from experimental device described previously (Roby et al., 1987), allows circulation of the perfusion medium controlled in solute composition, temperature, and pH through the living nodule sample. The partial oxygen pressure in the perfusion medium was established by bubbling mixtures of oxygen and nitrogen (either 21:79 or 1:99% O_2_:N_2_) into the medium reservoir. At various time, effectors were added into the perfusion medium. Each series of *in vivo* biological experiment has been performed at least five times.

^31^P NMR spectra were acquired at 202.47 MHz using a 500.16 MHz NMR spectrometer (Avance III, Bruker). For *in vivo* experiments, ^31^P NMR spectra were recorded for 36 min using a 10 mm ATMA broadband observe probe. A solution of 500 mM Hexamethylphosphoramide (HMPA, ref H3380, Sigma) contained in a concentric capillary provides the chemical shifts and intensity references for the ^31^P NMR spectra. 3,072 scans of 16 K data points were acquired with a 60° pulse angle, a spectral width of 14,204 Hz, acquisition time of 0.58 s and recycle delay of 0.1 s. Preliminary data processing was carried out with TOPSPIN 3.0 software (Bruker Biospin, Karlsruhe, Germany). Each Free Induction Decay (FID) was Fourier transformed (10 Hz line broadening), manually phased and baseline corrected. The resulting spectra were aligned by setting the HMPA signal to 30.73 ppm.

The resonance assignments were based on chemical shifts. ^31^P chemical shifts were determined according to (Rolin et al., 1989). Subcellular pH was estimated by the use of a standard reference curve of pH as a function of chemical shift, which was obtained according to the method of Roberts et al. (1980).

### Measurement of NR Activity

Tissue samples are ground with mortar and pestle in liquid nitrogen. The total proteins are extracted from 100 mg of powder using the following extraction buffer: 25 mM Tris HCl, pH 8.5, 1 mM EDTA, 20 μM FAD, 0.04% Triton X100, 10 μM NaMO_4_, 1 mM DTT, 20 µM L-transepoxysuccinyl-leucylamido-[4-guanidino]butane (E64), 2 mM phenylmethylsulfonyl fluoride (PMSF). The extracts are centrifuged (15,000 g, 15 min). The NR activity is measured by quantifying the NO_2_^−^ produced in the reaction mixture containing: the enzyme extract in 0.2 M HEPES, pH7.0, 15 mM KNO_3_, and 250 μM of NADH (Miranda et al., 2001). The reaction is stopped after 30 min by boiling the samples for 3 min at 100°C. The nitrite produced is measured using Griess reagent (1% sulphanilamide in 1 M HCl and 0.01% NEDD [N-1-naphthylethylenediamine dihydrochloride] in water) and measured at 540 nm. Soluble proteins are assayed according to the method of Bradford (1976). For each series of experiments, at least three independent biological replicates have been performed with three technical replicates per biological assay.

### RNA Isolation, Reverse Transcription and Gene Expressions

RNAs were isolated from 100 mg of frozen material ground in liquid N_2_ using the RNAzol following the manufacturer’s recommendations (Sigma-Aldrich). RNA quality was checked, and DNase treatment was carried out before the synthesis by GoScript reverse transcriptase (Promega) of the cDNAs. The RT-qPCR was made with Go-Taq qPCR master Mix kit according to manufacturer’s instructions (Promega). RT-qPCR data analysis was carried out using RqPCRBase, an R package working on R computing environment for analysis of quantitative real-time PCR data (Hilliou and Tran, 2013). The expression of the different genes was normalized against two housekeeping genes *Mtc27* (Van de Velde et al., 2006) and *Mta38* (del Giudice et al., 2011). The reference value ‘1’ was attributed to the first time when the cycle threshold (Ct) of the analyzed gene was significantly detectable. The data of comparative expression levels between genes are given on a logarithmic scale expressed as 40 − ΔC_T_, where ΔC_T_ is the difference in qRT-PCR threshold cycle number between the respective gene and the reference gene; the number 40 was chosen because PCR run stops after 40 cycles (Bari et al., 2006; Truong et al., 2015; Berger et al., 2020). RT-qPCR analyses were carried out in triplicate using the primers reported in **Table S2**. Three independent biological replicates have been performed.

### Enzymatic and HPLC Metabolite Analyses

Nodule metabolites (amino acids, organic acids, soluble sugars) were extracted by the alcoholic extraction method and resuspended in water as described in Brouquisse et al. (1991). Sucrose was determined enzymatically (Velterop and Vos, 2001) at 340 nm using a microplate spectrophotometer. Succinate and malate were determined by anion exchange HPLC (Dionex) with conductivity detection (Moing et al., 1998). Free amino-acids were analyzed by HPLC using the AccQ.Tag method from Waters (Milford, MA) with fluorescence detection (Moing et al., 1998). Three independent biological replicates have been performed with three technical replicates per biological assay.

### Extraction and Measurement of Nodule Adenine Nucleotides

Adenine nucleotides were extracted and measured as in Horchani et al. (2011). Adenine nucleotides were measured in a Xenius spectrofluorimeter-luminometer using the ATPlite one-step assay system (Perkin-Elmer) according to the manufacturer’s instructions. Three independent biological replicates have been performed with three technical replicates per biological assay.

### *β*-Glucuronidase Detection In Planta

For *β*-glucuronidase detection, nodulated roots of composite plants were incubated at −20°C for 1 h in a mixture “acetone:phosphate buffer (Na_2_HPO_4_/NaH_2_PO_4_ 0.1 M, pH 7.4)”, 90%:10% (v/v), then washed twice with the phosphate buffer and incubated 3 h to overnight at room temperature in the dark in phosphate buffer containing potassium ferricyanide (0.5 mM) and X-gluc (0;5 ng.ml^−1^). Nodulated roots or 80-µm-thick vibroslices, obtained with a HM560V Vibratome (Microm, http://www.microm.de) after embedding plant material in 4.5% low-melting-point agarose, were visualized with a Zeiss Axioplan II microscope (Carl Zeiss, http://www.zeiss.com) using dark-field optics. For each transformation experiment, 40 independent roots or nodules were transformed and individually analyzed for *β*-glucuronidase activity.

### Statistical Analyses

Statistical analyses were performed using Student *t*-test or one-way analysis of variance (ANOVA) followed by a Fisher test. Data were considered as significantly different when p < 0.05.

## Results

### NR Gene Expression During the Symbiosis Process

**Figure 1:**
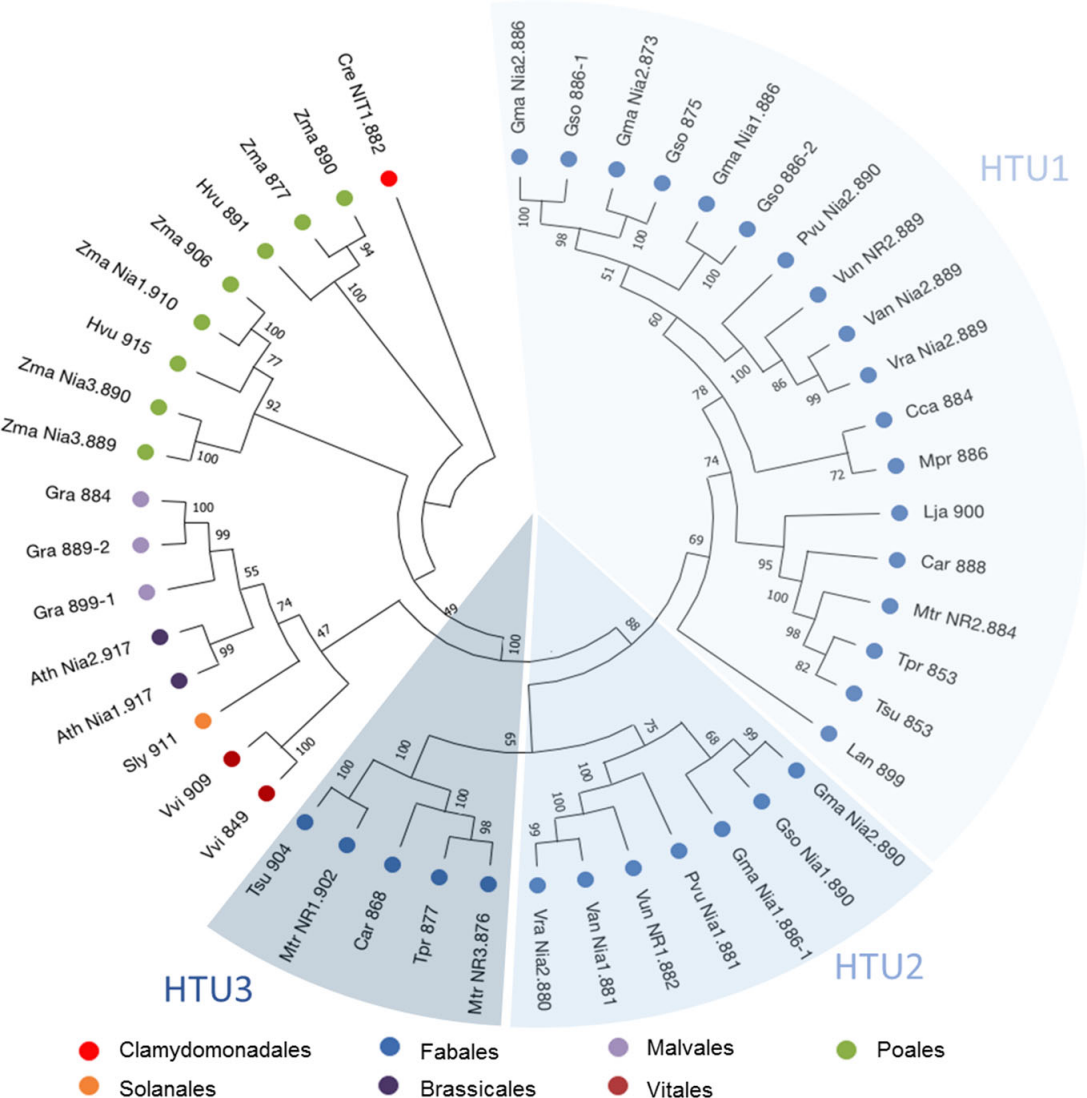
Phylogenetic tree of NR protein sequences. 47 nitrate reductase sequences from model plants or belonging to the Fabaceae family were used in this analysis. The tree was inferred by maximum likelihood (ML) with MegaX (Kumar et al., 2018). Percentages displayed next to each branch represent the number of tree replicates in which the associated taxa were assembled together when performing 1,000 bootstraps. The lengths of the branches do not represent a phylogenetic distance.

Research in three genomic and protein databases: NCBI (www.blast.ncbi.nlm.nih.gov), Phytozome (www.phytozome.jgi.doe.gov) and Uniprot (www.uniprot.org) revealed that all the plant genomes studied code for one to six NR sequences, with 76% of the species having between one and two NR (**Table S1**). Among the genomes of the Fabales group in **Table S1**, only three species seem to have more than two NR sequences: *Glycine max* and *Glycine soja*, with five and four sequences respectively and *M. truncatula* with three sequences. The three *M. truncatula NR* genes (Puppo et al., 2013; Roux et al., 2014), named Mt*NR1*, Mt*NR2*, and Mt*NR3*, respectively encode proteins of 902, 884, and 876 amino acids (**Figure S1A**). The three *NR* genes exhibit strong similarities (>70%) to each other and exhibit similar structure with four exons and three introns of different sizes (**Figure S1B**). Both Mt*NR1* and Mt*NR3* are closely present in chromosome 3, while Mt*NR2* is present in chromosome 5. To study the phylogenetic relationships operating on the NR sequences, a phylogenetic tree using NR from the Fabales family and different model plants was inferred (**Figure 1**). Forty seven non-redundant protein sequences from 21 species and belonging to the Solanales, Fabales, Brassicales, Malvales, Vitales, and Poales families were used (**Table S1**). The most striking result is the separation of the Fabales sequences into three distinct Hypothetical Taxonomic Units (HTU), named HTU1, HTU2, and HTU3 (**Figure 1**). MtNR2 is in the largest unit HTU1, whereas Mt*NR1* and Mt*NR3* are both in HTU3. For species with more than one sequence, we observe a distribution of these sequences over two HTUs.

**Figure 2:**
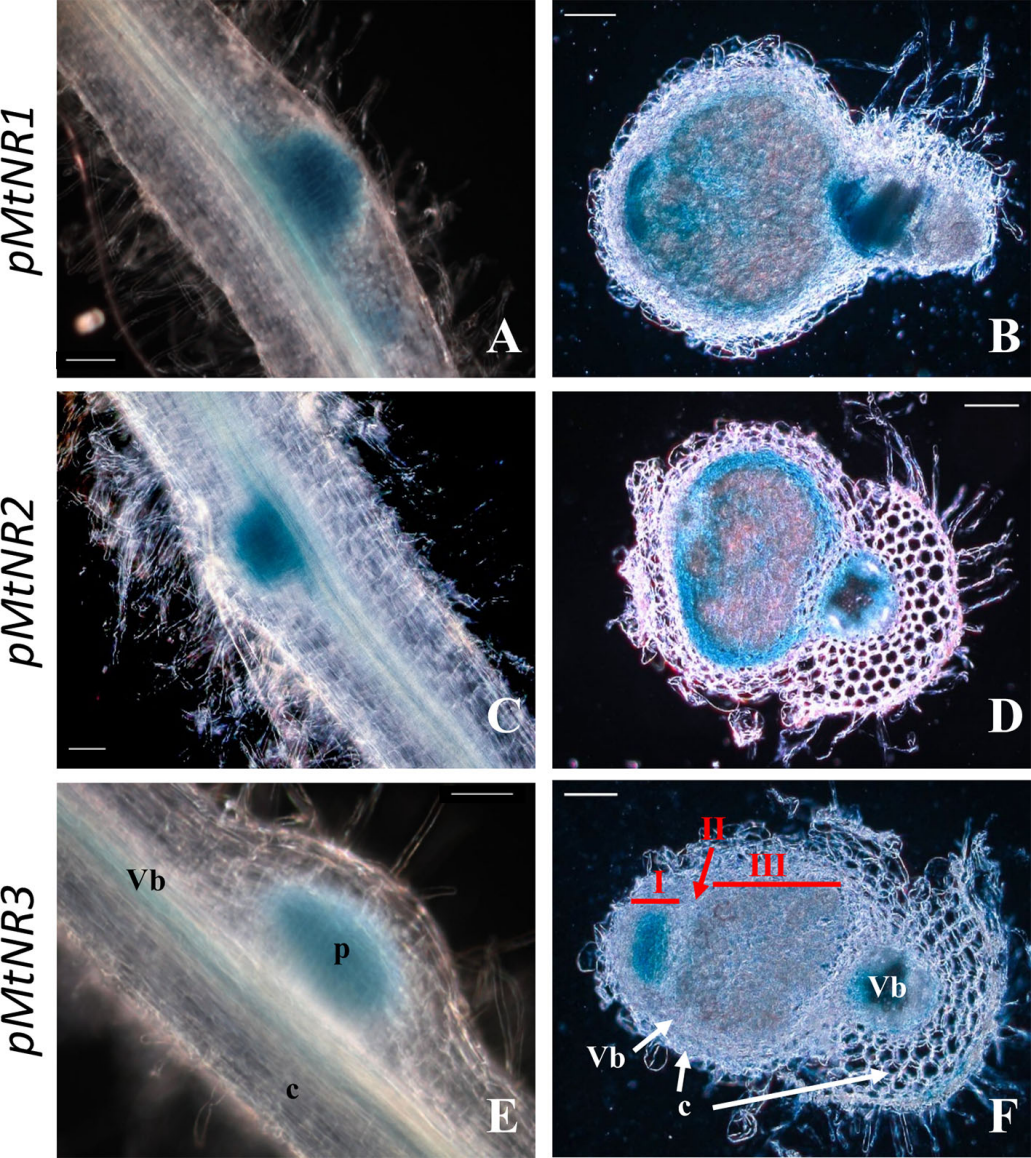
Histochemical localization of *MtNRs* expression in *Medicago truncatula* roots. Localization of GUS activity in transgenic *M. truncatula* roots expressing the gusA reporter gene under the control of a 1.65 Kb *MtNR1* promoter fragment **(A, B)**, of a 1.7 kb *MtNR2* promoter **(C, D)** and of a 1.55 kb *pMtNR3* promoter **(E, F)**. Whole root segment 4 dpi with *S. meliloti* **(A, C, E)** and longitudinal section of a 2 wpi old nodules **(B, D, F)** were stained for 3, 5, or 16 h with X-gluc for the GUS activity for *pMtNR2, pMtNR1*, or *pMtNR3* respectively. Zones I, II, and III of the nodule are represented in red in picture F. p, nodule primordium; Vb, vascular bundles; c, cortex. Scale bars, 50 µm for **(A, C, E)**; 100 µm for **(B, D, F)**.

As reported in the Affymetrix data (**Figure S2**), both Mt*NR1* and Mt*NR2* are significantly expressed in the roots and nodules, but Mt*NR3* is specifically expressed in the nodules. More precisely, the histochemical detection of GUS activity under the control of *NR* promoters at 4 dpi shows that the three NR genes are expressed in young developing nodules, *i.e.* in the controlled area (a block of cells contituted by pericycle, endodermis and dividing cortical cells, Xiao et al., 2014) of nodule primordium (**Figures 2A, C, E**). In 14 dpi-old nodules, Mt*NR1* expression is detected in the whole nodule (**Figure 2B**). *NR2* expression is also detected in the three zones but also at the periphery of the nodule in the vascular bundle (**Figure 2D**). In the case of Mt*NR3*, GUS staining appears mainly at the level of zones I and II and very slightly in zone III (**Figure 2F**). These results are consistent with Symbimics data (Roux et al., 2014; **Table 1**) which report a localization of the expression of the three NR genes close to that which we observe in 14 dpi-old nodules.

**Table 1:** Access code and Symbimics expression of *Medicago truncatula* NR and NO-producing enzyme genes.

Genes	Code Affymetrix	Code gene Mt4.0	Code gene Mt5.0	Mt20120830-LIPM	DESEQ MEAN				
					FI	FIID	FIIP	IZ	ZIII
NR1	Mtr.42446.1.S1_at	Medtr3g073180	MtrunA17Chr3g0115151	Mt0006_00730	20.7	89.1	21.3	193.7	693.5
NR2	Mtr.10604.1.S1_at	Medtr5g059820	MtrunA17Chr5g0424491	Mt0008_10301	156.6	166.9	266.7	712.9	843
NR3	Mtr.31448.1.S1_at	Medtr3g073150	MtrunA17Chr3g0115131	Mt0006_00731	1.3	0.5	1.3	0	1.4
XDH1	Mtr.23395.1.S1_at	Medtr2g098030	MtrunA17Chr2g0328851	Mt0016_10367	80	52.3	37.3	39.2	52.7
AAO3	Mtr.29357.1.S1_at	Medtr5g087390	NC	Mt0010_10456	29.05	22.75	10.23	12.61	25.35
IAO3	Mtr.42638.1.S1_at	Medtr5g087410	NC	Mt0010_10457	4.95	9.36	4.05	19.45	62.19
NOFNiR1	Mtr.40060.1.S1_at	Medtr2g035460	MtrunA17Chr2g0296891	Mt0101_10060	43	29.2	4.1	18.8	125.4
NOFNiR2	Mtr.10348.1.S1_at	Medtr2g035470	MtrunA17Chr2g0296881	Mt0101_10060	43	29.2	4.1	18.8	125.4
NOFNiR3	Mtr.33463.1.S1_s_at	Medtr2g035480	MtrunA17Chr2g0296871	Mt0101_10060	43	29.2	4.1	18.8	125.4
SOX1	Mtr.28230.1.S1_at	Medtr6g023975	MtrunA17Chr6g0459641	Mt0033_10312	53	55.7	60.2	137.4	121.9
SOX2	NC	Medtr7g033410	MtrunA17Chr7g0227151	Mt0044_10132	3.3	1.5	2.6	4.6	9.2

Affimetrix gene codes were on (https://mtgea.noble.org/v3/), Mt4.0 gene codes were on (https://www.jcvi.org/medicago-truncatula-genome-database), Mt5.0 gene codes were on (https://medicago.toulouse.inra.fr/MtrunA17r5.0-ANR/), Symbimics data were in Roux et al. (2014). NC, no code available; DESEQ MEAN corresponds to the expression value of the RNA-sequencing analysis in the different zones of the nodule; FI, meristematic zone; FIID, distal infection zone; FIIP, proximal infection zone; IZ, interzone; ZIII, N_2_-fixing zone. Shades of red highlight the highest expression value for each gene. NR, nitrate reductase; XDH, xanthine dehydrogenase; AAO, abscissic aldehyde oxidase; IAO, indole aldehyde oxidase; NOFNiR, NO forming nitrite reductase; SOX, sulfite oxidase.

**Figure 3:**
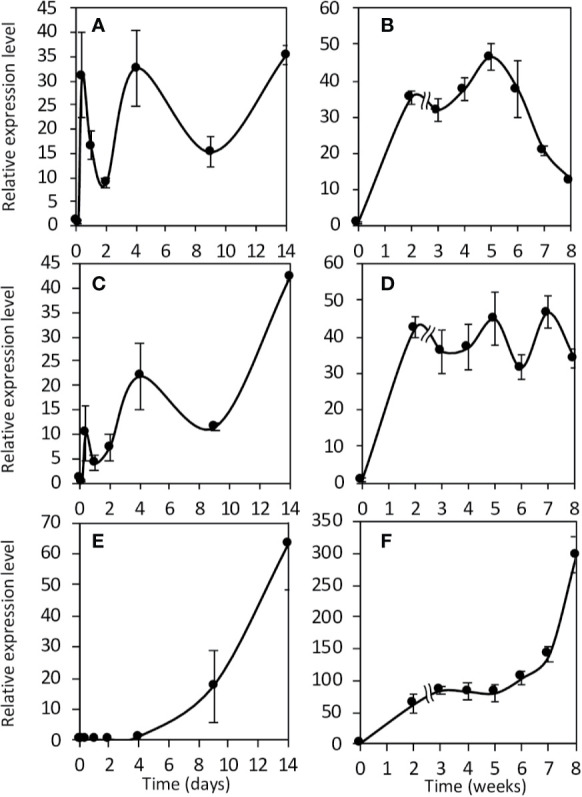
Expression of *Medicago truncatula NR* genes during the symbiotic process. Expression analysis of the NR genes in roots and nodules during the symbiotic process. Short term kinetic 14 dpi **(A, C, E)**, long term kinetic 8 wpi **(B, D, F)**. Expression of NR1 **(A, B)**, NR2 **(C, D)** and NR3 **(E, F)**. Data are means ± SE of three biological replicates. Each measure was realized in three technical replicates. dpi, day post inoculation; wpi, week post inoculation.

**Figure 4:**
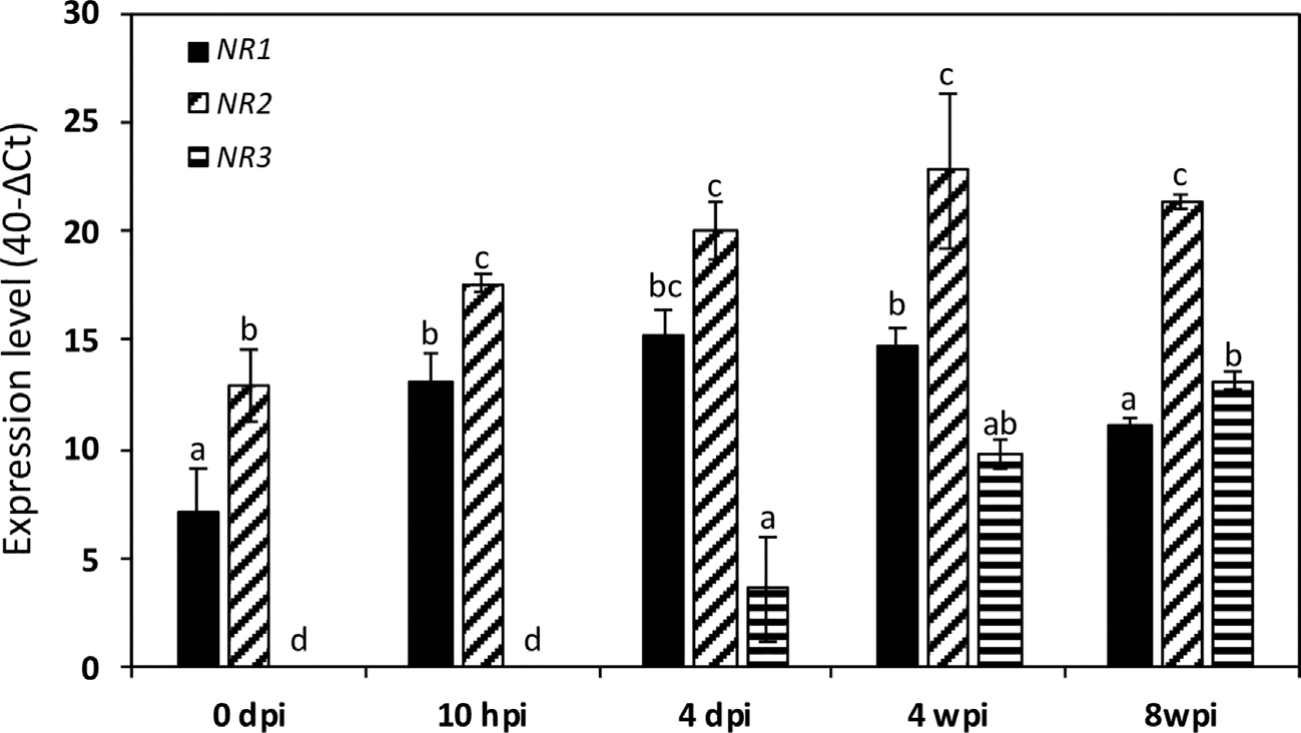
Expression of *Medicago truncatula NR* genes at various times of the symbiotic process. The data are drawn from the same experiments as those presented in **Figure 3**. Values followed by different letters are significantly different according to one-way ANOVA analysis followed by a Fisher test (P<0.05). dpi, day post inoculation; hpi, hour post inoculation; wpi, week post inoculation.

Then, we investigated their expression kinetics throughout the symbiotic process. To this end, we used two types of *M. truncatula* cultures: a short-term culture from 0 to 14 dpi and a long-term culture from 0 to 8 wpi. Both Mt*NR1* and Mt*NR2* are expressed at a significant level in non-inoculated roots (**Figures 3** and **4**). After inoculation with *Sinorhizobium meliloti*, Mt*NR1* expression significantly increases (30- to 45-fold) and exhibits three expression peaks at 10 hpi, 4 dpi, and 5 wpi (**Figures 3A, B**). Similarly, Mt*NR2* expression increases with two peaks at 10 hpi and 4 dpi and then reaches a plateau between 3 and 8 wpi (**Figures 3C, D**). Mt*NR3* expression is detected only from 4 dpi, increases to reach a plateau between 3 and 5 wpi, and then strongly increased (up to 300 times) after 6 wpi at the nodule senescence (**Figures 3E, F**). The expression level of *NR* as compared to each other, before inoculation and at four time-points in the symbiosis, is reported in **Figure 4**. Several features emerged from this analysis. 1) Mt*NR2* is the most highly expressed *NR* gene (30 to 100 times more than *NR1*) throughout the whole process. 2) The three *NR* genes are significantly expressed in N_2_-fixing nodules. 3) Whereas the expression of Mt*NR1* decreases at the onset of nodule senescence, the expression of Mt *NR3* clearly increases and exceeds that of Mt*NR1* in 8 wpi nodules. By comparison with the other enzymes potentially involved in the synthesis of NO such as abscissic aldehyde oxidase (AAO), indole aldehyde oxidase (IAO), NO forming nitrite reductase (NOFNiR), sulfite oxidase (SOX), and xanthine dehydrogenase (XDH), it may be noted that the expression of *MtNR1* and *MtNR2* is significantly stronger than that of the other genes (**Figure S2**).

### NR Activity and NO Level During the Symbiotic Process

**Figure 5:**
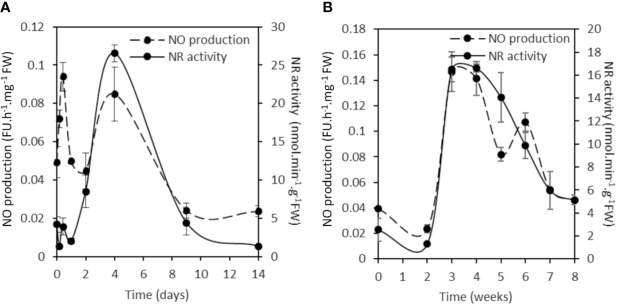
NR activity and NO production during the *Medicago truncatula* symbiosis. Short term kinetic 14 dpi **(A)**, long term kinetic 8 wpi **(B)**. The nitrate reductase activity is expressed in nmol per min per g of fresh weight. The fluorescence intensity of the NO production was measured using the DAF-2 fluorescent probe. Data are means ± SE of three biological replicates. Each measure was realized in three technical replicates. dpi, day post inoculation; FU, fluorescence unit; wpi, week post inoculation.

**Figure 6:**
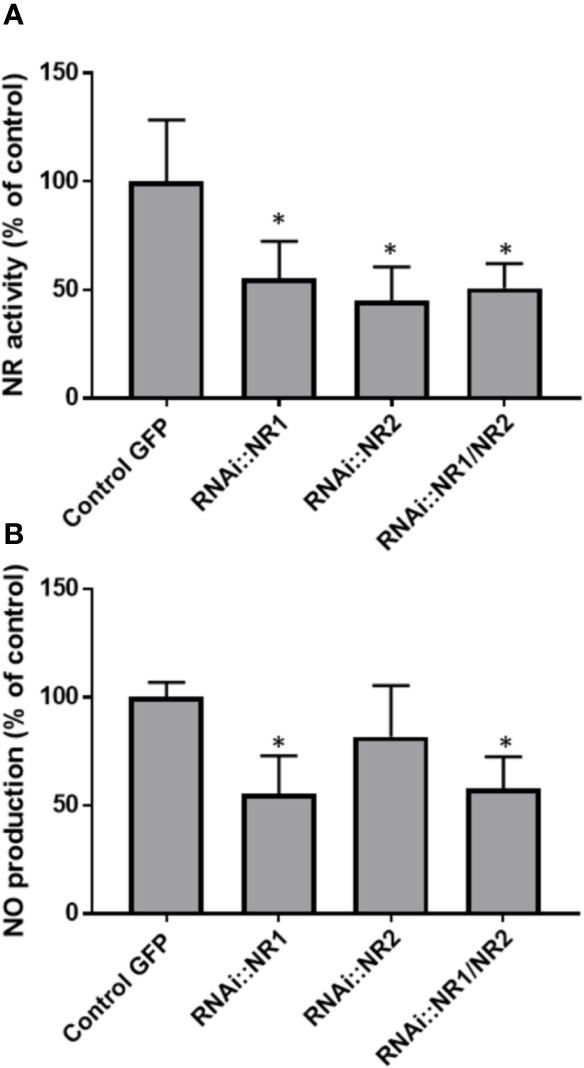
NR activity and NO production in NR RNAi transformed *Medicago truncatula* nodules. **(A)**, NR activity is expressed in nmol per min per g of fresh weight. **(B)**, The fluorescence intensity of the NO production was measured using the DAF-2 fluorescent probe. Data are means ± SE of three biological replicates. Each measure was realized in three technical replicates. Asteriks * indicate statistical difference when compared with the control GFP (transgenic roots transformed with empty pK7GWIWG5D(II) vector selected on GFP marker) at P < 0.05 according to Student’s *t* test. FU, fluorescence unit.

To assess the contribution of NR in NO production, the total NR activity was analyzed and compared to the NO production level during the symbiotic process (**Figure 5**). NR activity in non-inoculated roots is close to 4.2 ± 0.9 nmol.min^−1^.g^−1^FW. Following inoculation, NR activity exhibits a significant and reproducible 60–70% decrease within 4 hpi before returning close to its initial value at 10 hpi. After a new decrease at 24 hpi, NR activity strongly increases to 26.5 ± 1.1 nmol.min^−1^.g^−1^FW at 4 dpi, strongly decreases between 9 and 14 dpi, and then increases again to peak at 3–5 wpi. After the onset of nodule senescence, NR activity decreases to reach 5.1 ± 0.1 nmol.min^−1^.g^−1^ FW at 8 wpi. As far as it is concerned, NO production level shows three transient production peaks, at 10 hpi, 4 dpi, and 3–4 wpi (**Figures 5** and **S3**; Berger et al., 2020). As a whole, the pattern of NR activity follows that of Mt*NR1* and *2* gene expression (**Figure 3**) and clearly shows a parallel with the production of NO (**Figure 5**). It particularly fits with the expression of Mt*NR1* and Mt*NR2* during the three first weeks of the symbiosis and with that of Mt*NR1* between 3 and 8 wpi, suggesting that NR1 is playing a major role in NO production. To check this hypothesis, a RNAi strategy was used. *M. truncatula* RNAi on the Mt*NR1*, Mt*NR2*, and double Mt*NR1-2* genes were constructed under the control of the zone III-specific promoter NCR001 (Mergaert et al., 2003; Horchani et al., 2011). Four wpi-old nodules were collected and analyzed for NR activity and NO production. As compared to the control, NR activity is decreased by 47 and 56%, respectively, in the RNAi::*NR1* and *NR2* nodules (**Figure 6A**), but the double RNAi*::NR1-2* does not make it possible to further reduce the total NR activity. Regarding NO production, it drops by 45% in RNAi::*NR1* nodules, but only by 18% in RNAi::*NR2* nodules (**Figure 6B**). The decrease in NO production in the nodules of double RNAi::*NR1-2* is of the same order of magnitude as that observed in RNAi::*NR1* nodules. These results clearly show that the decrease in NR activity is accompanied by a fall in NO production and that this fall is particularly related to NR1 activity rather than NR2.

### Involvement of NR Activity in NO Production and Energy Regeneration During Nodule Development

**Figure 7:**
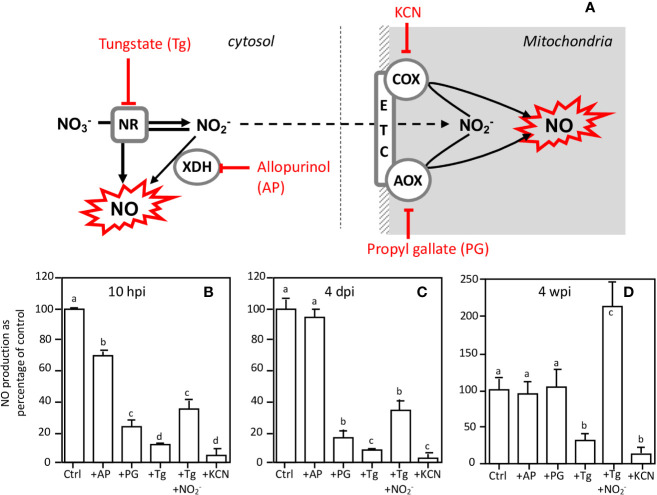
Effects of inhibitors on NO production at various times of the *Medicago truncatula* symbiosis. **(A)**, schematic representation of inhibitor targets. NO production of 10 hpi-old **(B)** and 4 dpi-old **(C)** roots and of 4 wpi-old nodules **(D)** was measured after 4 h in the presence of either 1 mM tungstate (Tg), 1 mM allopurinol (AP), 1 mM propyl gallate (PG), 1 mM nitrite (NO_2_^−^), or 300 μM KCN. NO production is expressed as the percentage of the control values. Data are means ± SE of three biological replicates. Each measure was realized in three technical replicates. Values followed by different letters are significantly different according to one-way ANOVA analysis followed by a Fisher test (P < 0.05). dpi, day post inoculation; hpi, hour post inoculation; wpi, week post inoculation. AOX, alternative oxidase; COX, cytochrome oxidase; ETC, electron transfer chain; NR, nitrate reductase; XDH, xanthine dehydrogenase.

The concomitance of the peaks of the NR expression/activity and those of NO production suggests that NRs are involved in the production of NO. Thus, using an inhibitor approach, we investigated the participation of NR and other potential NO sources such as xanthine dehydrogenase and mitochondrial electron transport chain (ETC) in NO production in 10 hpi and 4 dpi roots and in 4 wpi nodules. As reported in **Figure 7**, NO production is 95% inhibited by KCN, used as a negative control. Allopurinol (AP), a specific inhibitor of xanthine dehydrogenase (XDH), moderately inhibits NO production (by 28%) in 10 hpi roots, whereas it is without effect in 4 dpi roots and 4 wpi nodules. In both 10 hpi and 4 dpi roots, propyl gallate (PG), an inhibitor of the mitochondrial alternative oxidase (AOX), inhibits NO production by 70–90%, indicating that the mitochondria are involved in this reaction. However, PG is ineffective in 4 wpi nodules. It can be noted that PG is also an inhibitor of polyphenol oxidases (Lin et al., 2013), but that the latter are not known to be directly or indirectly involved in the production of NO. The effects of PG on NO production can therefore be attributed to AOX inhibition. Interestingly, tungstate (Tg), a NR inhibitor, inhibits NO production by 88, 92, and 60% respectively in 10 hpi roots, 4 dpi roots, and 4 wpi nodules, and this inhibition is either partially (10 hpi and 4 dpi roots) or totally (4 wpi nodules) relieved by the addition of nitrite, the reaction product of NR (**Figure 7**). These data suggest that NR and ETC are involved in NO production.

To further investigate the potential involvement of NR and NO in energy metabolism, we analyzed the energy state (*i.e.* the ATP/ADP ratio) in 10 hpi and 4 dpi roots and in 4 wpi nodules after a 4 h treatment in the presence of NR effectors (**Table 2**). In control and NO_2_^−^-treated roots and nodules, ATP/ADP ratio is high, indicating that ATP-regenerating processes are not limited. In the presence of Tg, the ATP/ADP ratio is significantly decreased only in 4 wpi nodules, indicating that the inhibition of NR partially affects the energy state in mature nodules, but not in 10 hpi and 4 dpi roots. This decrease in the ATP/ADP ratio is not observed when 4 wpi nodules are incubated in the presence of both Tg and NO_2_^−^, which means that the supply of NO_2_^−^ makes it possible to maintain the nodule energy state.

**Table 2:** Effects of NR effectors on ATP/ADP ratio in *Medicago truncatula* roots and nodules.

		ATP/ADP ratio		
		10 hpi	4 dpi	4 wpi
Control		6.1 ± 1.1	6.3 ± 1.7	6.8 ± 1.2
+1 mM NO_2_^−^		5.7 ± 0.9	4.7 ± 0.4	6.4 ± 0.7
+1 mM Tg		5.2 ± 0.5	5.6 ± 3.3	4.4 ± 0.4 *
+1 mM NO_2_^−^ +1 mM Tg		4.9 ± 0.6	4.5 ± 1.0	5.5 ± 0.7

Either 10 hpi roots, 4 dpi roots, or 4 wpi-old nodules were incubated for 4 h in the presence of either 1 mM nitrite (NO_2_^−^), 1 mM tungstate (Tg), or both. Data are the means ± SE of three (10 hpi, 4 dpi) and four (4 wpi) biological replicates. Each measure was realized in three technical replicates. Asterisk * indicates statistical difference with the control at the same time point at P < 0.05 according to Student’s t test. dpi, day post inoculation; hpi, hour post inoculation; wpi, week post inoculation.

Considered together, these results indicate that 1) both the NR and the mitochondrial ETC are involved in the production of NO, probably *via* the reduction of nitrate to nitrite by NR and the subsequent reduction of nitrite to NO at the mitochondrial ETC level, and 2) NR activity is linked to energy regeneration processes in mature nodules, but not during the first steps of the symbiosis.

### Involvement of NR in Carbon and Nitrogen Metabolism in Mature Nodules

**Figure 8:**
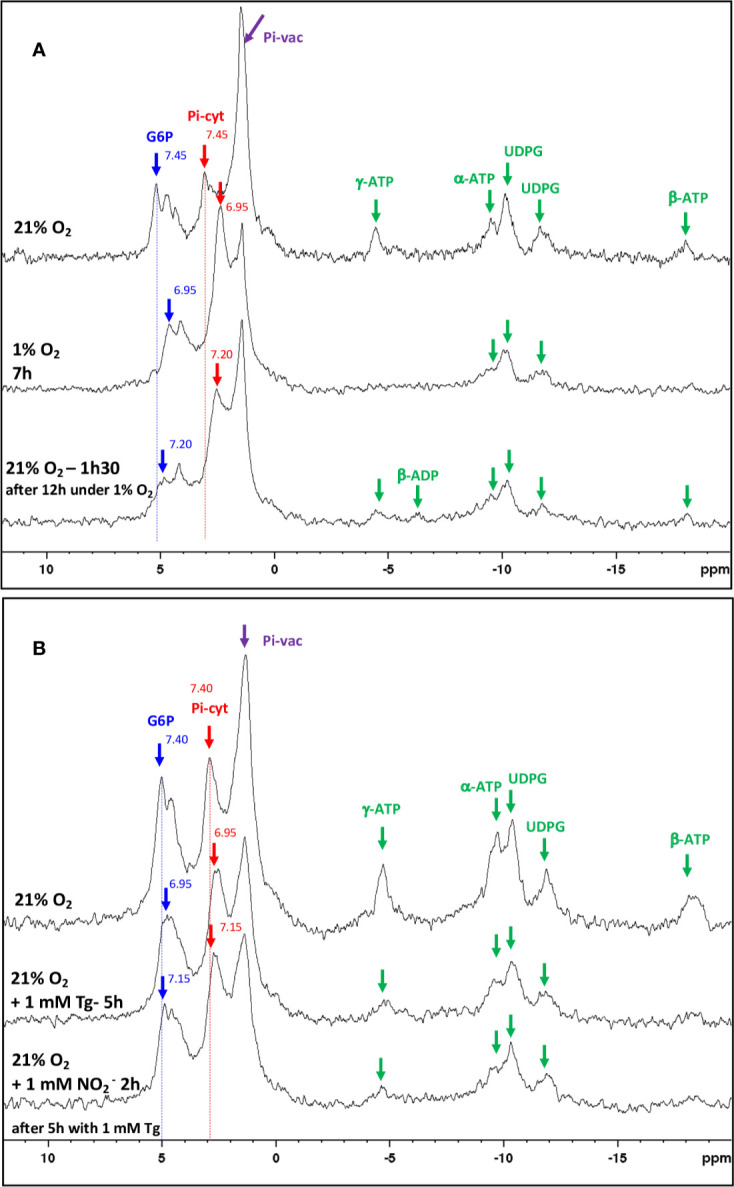
*In vivo* ^31^P-NMR study of metabolic transitions induced in *Medicago truncatula* nodules. **(A)** Proton-decoupled ^31^P-NMR spectra of nodules in normoxia at 21°C, perifused with a nutritive medium at pH 6.0 with successively 21% O_2_ (control), 1% O_2_, and then 21% O_2_. For each line, duration of the treatment is indicated up to the end of the spectrum. **(B)** Proton-decoupled ^31^P-NMR spectra of nodules in normoxia at 21°C, perifused with a nutritive medium at pH 6.0 containing successively 21% O_2_ (control), 1 mM tungstate (Tg) for 5 h, and then 1 mM nitrite (NO_2_^−^) for 2 h. For each line, duration of the treatment is indicated up to the end of the spectrum. Each spectrum series is drawn from a representative experiment of eight **(A)** and five **(B)** biological replicates. The values indicated next to the G6P and Pi-cyt resonances correspond to the cytosolic pH values. Exponential apodization and zero filling. Pi, inorganic phosphate; G6P, Glucose-6-phosphate; Pi-cyt, cytoplasmic Pi; Pi-vac, vacuolar Pi; UDPG, UDP-glucose.

Based on the above results, it appeared important to investigate more precisely the role of NR activity in the energetic metabolism of mature nodules. To this end, using a perifusion system adapted to NMR spectrometer (Roby et al., 1987), we first followed the effects of *in vivo* transition from normoxia to hypoxia, and *vice versa*, on the energy metabolism of 4 wpi nodules. **Figure 8A** displays typical ^31^P spectra obtained with living *M. truncatula* nodules during normoxia–hypoxia–normoxia transition experiments. The main resonance peaks are attributed to glucose-6-phosphate (G6P), cytosolic and vacuolar inorganic phosphate (Pi-cyt and Pi-vac, respectively), uridine di-phospho-glucose (UDPG), and to *α*, *β* and *γ* ATP. Transition from 21% O_2_ (normoxia) to 1% O_2_ (hypoxia) leads to an important reduction in ATP content and a significant acidification of cytoplasmic pH from 7.45 to 6.95 after 7 h of hypoxia, as measured by the shift of G6P and Pi-cyt resonances (**Figure 8A**). Back transition from 1 to 21% O_2_ is accompanied by an increase in ATP and a progressive return of cytoplasmic pH to more alkaline values (7.2). In a second series of experiments, nodules were incubated at 21% O_2_ in the presence of 1 mM Tg (**Figure 8B**). After 5 h of Tg treatment, ATP level decreases by a factor 2.5, and cytoplasmic pH decreases from 7.4 to 6.95, indicating a decrease in energy state and an acidification of the nodules. The addition of 1 mM NO_2_^−^ triggers an increase in cytosolic pH to 7.15, indicating a progressive recovery of the cell metabolism (**Figure 8B**). Thus, the inhibition of NR by Tg and its subsequent relief by NO_2_^−^ partially mimicks “normoxia–hypoxia–normoxia” transitions and support the involvement of NR in the energy metabolism of nodules.

**Figure 9:**
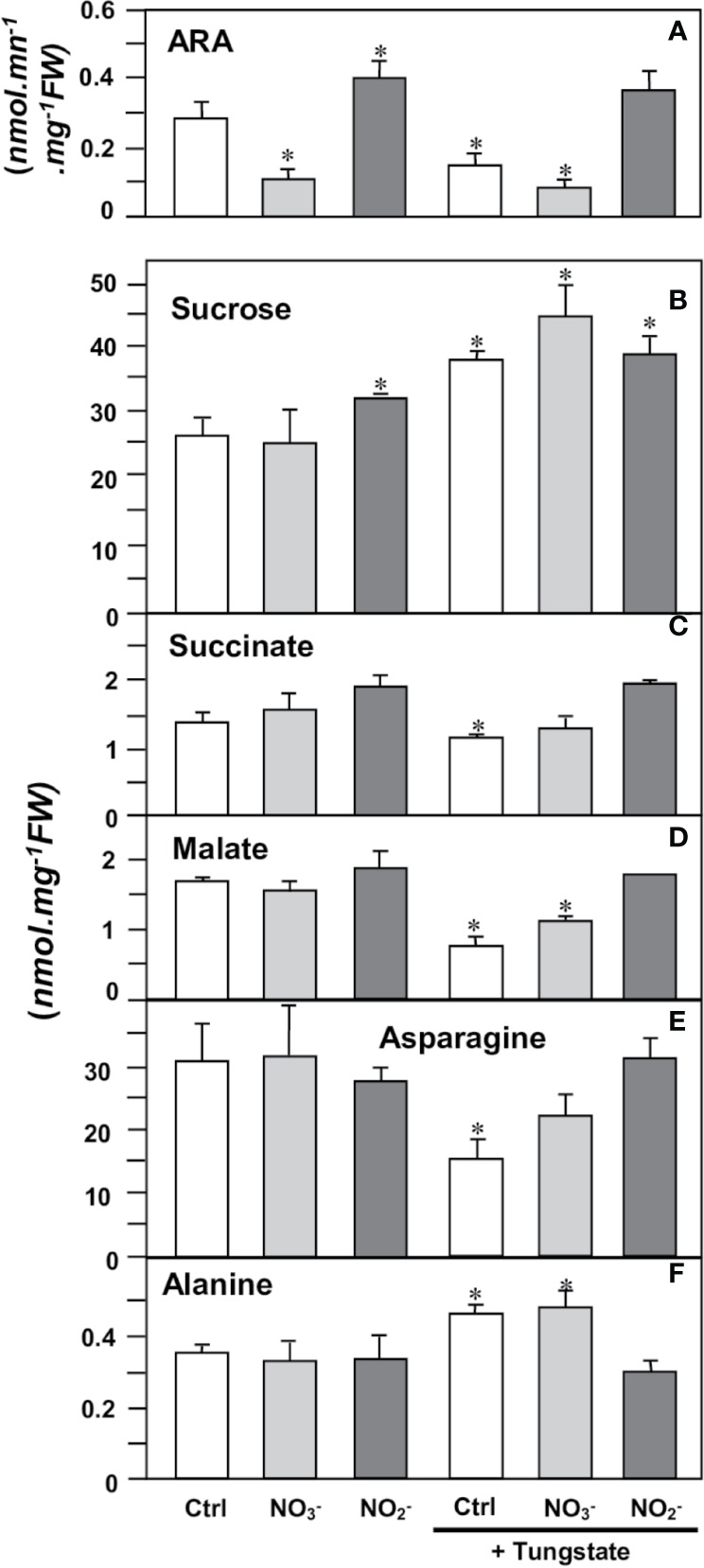
Effects of NR effectors on nitrogenase activity and metabolite contents in *Medicago truncatula* nodules. 4 wpi-old nodules were incubated for 4 h in the presence of either 10 mM nitrate (NO_3_^−^), 1 mM nitrite (NO_2_^−^), or 1 mM tungstate (Tg). **(A)**, nitrogenase activity (estimated as ARA); **(B)**, sucrose; **(C)**, succinate; **(D)**, malate; **(E)**, asparagine; **(F)**, alanine. Data are means ± SE of three biological replicates. Each measure was realized in three technical replicates. Asterisks * indicate significant difference at P<0.05, when compared with the control (Ctrl) according to Student’s *t* test.

Then, we investigated the potential involvement of NR in the N_2_-fixing metabolism. To this end, mature nodules were incubated for 4 h in either the presence, or the absence, of 1 mM Tg, 10 mM NO_3_^−^ and 1 mM NO_2_^−^. As reported in **Figure 9**, the nitrogenase activity, as measured by its acetylene reducing activity (ARA), is inhibited in the presence of either NO_3_^−^ or Tg (or both) but is unaffected by the presence of NO_2_^−^, indicating that NR activity supports N_2_ fixation. The changes in the content of various metabolites related to carbon and nitrogen metabolism of the nodules are consistent with this hypothesis. Thus, following NR inhibition by Tg (either in the presence or absence of NO_3_^−^), sucrose content increased, indicating a slowdown of its consumption by the nodules (**Figure 9B**). As a consequence, succinate and malate contents decreased (**Figures 9C, D**), indicating a lower supply of carbon nutrients to bacteroids, and asparagine content decreased (**Figure 9E**) as a result of the shortage of carbon substrate supply and ARA inhibition. Finally, alanine, which is a marker of hypoxia in plant tissues (Gibbs and Greenway, 2003), was found to increase in the presence of Tg (**Figure 9F**), indicating that NR inhibition mimics a hypoxia situation. The presence of NO_2_^−^ reverses the effects of Tg (**Figure 9**).

## Discussion

### Medicago truncatula NRs Are Involved in NO Production

The main objective of this study was to test the hypothesis that NR regulates NO production and controls N_2_-fixing metabolism during the *M. truncatula*–*S. meliloti* symbiosis.

The Medicago genome encodes three *NR* genes, Mt*NR1*, Mt*NR2*, and Mt*NR3* (Puppo et al., 2013; Roux et al., 2014). Whereas most of the species analyzed possess between one and two sequences of NR, *M. truncatula* have three NRs. Mt*N3* and Mt*NR1* are both located on chromosome 3 separate from only 20 kb and are grouped in the same HTU on the phylogenetic tree, elements which are in favor of an event of duplication of Mt*NR1* to give birth to Mt*NR3*. This exhaustive search for NR sequences conducted to identify two potential orthologs to Mt*NR3* in species closed to *M. truncatula* such as *Trifolium pratense* and *Cicer arietanum*. However, we did not identify three NR sequences in *T. pratense* and *C. arietanum*. Mt*NR3* has been shown to be specifically expressed in symbiotic nodules, excluding other organs of the plant (http://mtgea.noble.org/v3/). Its expression from 4 dpi (**Figures 3** and **4**) and location in the nodule primordium as well as in zones I and II of the nodule (**Figure 2**, **Table 1**) suggest that MtNR3 plays a role in cell division and infection processes during nodule organogenesis. Similarly, the sharp increase in its expression beyond 6 wpi (**Figure 3F**) suggests that NR3 also plays an important role in nodule senescence process that will be worth investigating. However, its low expression level during symbiosis (**Figure 4**) and its expression pattern, very different from the NO production once (**Figures 3** and **5**), do not support, *a priori*, a potential involvement of NR3 in the production of NO, pointing to a specific role of this NR in nodule aging.

Mt*NR1* and Mt*NR2* correspond to inducible and/or constitutive forms that have been found in other higher plants such as *A. thaliana* and *G. max* (Santucci et al., 1995). Our results show that three concomitant peaks of Mt*NR1*/*NR2* expression and NR activity can be considered over the period analyzed (**Figures 3****–****5**): i) during the first hours of the symbiotic interaction at10 hpi; ii) during the early development of the nodule at 4 dpi; and iii) when the nodule becomes mature around 3–4 wpi. The most salient feature of this study is the parallel that can be drawn between the expression of Mt*NR1* and Mt*NR2* (**Figure 3**), the total NR activity (**Figure 5**), and the production of NO (**Figure 5**). During the infection phase, at 10 hpi, the increase in Mt*NR1* and Mt*NR2* expression is accompanied by a slight increase in NR activity, while the production of NO increases by a factor of 2, suggesting that NRs are probably not the only sources of NO. In contrast, later in the symbiotic process (at 4 dpi and 3–4 wpi), the correlation between gene expression, NR activity, and NO production suggests that NO production is directly related to NR. Moreover, the spatiotemporal expression of Mt*NR1* and Mt*NR2* (**Figure 2**) corresponds to the localization of NO production reported to occur in the nodule primordium (del Giudice et al., 2011) and in the N_2_-fixing zone of the mature nodule (Baudouin et al., 2006). While keeping in mind that Mt*NR2* is much more expressed than Mt*NR1*, these observations strongly suggest that both NR1 and NR2 are involved in the production of NO during the symbiotic process. In a recent study dealing with the role of *M. truncatula* phytoglobins (Phytogbs) during N_2_-fixing symbiosis we found that *Phytogb1.1* exhibits an expression pattern similar to that of NO production and is involved in its regulation during the different stages of the symbiosis (Berger et al., 2020). Thus, regardless of the potential mechanisms of post-translational regulation of Phytogbs and NRs activities, our results indicate that NRs probably work in coordination with Phytogb1.1 to regulate the level of NO at each stage of the symbiotic process.

The use of inhibitors of different NO sources (**Figure 7**) supports the hypothesis that at 10 hpi, 4 dpi, and 4 wpi, NRs are either directly, or indirectly, in combination with the mitochondrial ETC, involved in the production of NO. In fact, at these three stages the production of NO is inhibited by Tg. The use of Tg to inhibit NR activity has been successfully used in mature *M. truncatula* nodules to demonstrate the involvement of NR in NO production (Horchani et al., 2011). However, Tg is known to inhibit other molybdoenzymes than NR, including XDH, aldehyde oxidase, sulfite oxidase, and NO forming nitrite reductase (NOFNiR) (Mendel and Hänsch, 2002; Xiong et al., 2012, Chamizo-Ampudia et al., 2016), and the possibility that the Tg-dependent inhibition of NO production is linked to the inhibition of one of these other enzymes cannot be excluded. Notwithstanding the stability and the specific activity of these enzymes in the root and nodule cells, the weak expression level of their genes remains much lower than those of Mt*NR1* and Mt*NR2* (**Table 1**, **Figure S2**), and one can reasonably think that NRs remain the major molybdoenzymes in cells. The use of the inhibitor allopurinol (**Figure 7**) shows that XDH, of which expression remains significant during the symbiotic process (**Table 1**, **Figure S2**), is perhaps partially involved in the production of NO during the first hours of symbiosis, but neither during nodule organogenesis nor in the N_2_-fixing nodule. Furthermore, the preferential expression of XDH in the infection zone of the nodules (**Table 1**) reinforces the hypothesis of its involvement in the defense responses which occur at the beginning of the symbiotic process. XDH is a peroxisomal enzyme capable of producing NO^•^ and superoxide anion (O_2_^•−^) that can complex together to give peroxynitrite, ONOO^−^ (del Río et al., 2004). ONOO^−^ is known to play a key role in the induction of defense responses, particularly *via* tyrosine nitration of proteins (Saito et al., 2006), which is consistent with the involvement of NO production in defense responses at 10 hpi.

Otherwise, the partial or total reversion of Tg-dependent inhibition of NO production by nitrite (**Figure 7**) means that NRs at least produce the nitrite necessary for the production of NO. Indeed, the inhibition of NO production by mitochondrial ETC inhibitors (**Figure 7**) supports the hypothesis that NR activity is indirectly involved in NO production *via* the reduction of nitrate to nitrite and the subsequent nitrite reduction to NO by mitochondrial ETC. This indicates that from the beginning of the symbiotic process, a major part of NO is produced *via* a nitrite reducing pathway.

We should also keep in mind that NO in the nodule is not produced only by the plant partner. Indeed, the bacterial partner was shown to produce from 33 to 90% of NO in *M. truncatula* (Horchani et al., 2011) and soybean (Sanchez et al., 2010) nodules, respectively. If the bacterial denitrification pathway, including the periplasmic nitrate reductase (Nap) and nitrite reductase (Nir), has been described as the main enzymatic source of NO, additional genes encoding putative nitrate and nitrite reductase (called narB and NirN, respectively) have been recently identified that could also participate indirectly in NO synthesis (Ruiz et al., 2019). How the regulatory systems of the plant and the bacterial partners are coordinated to produce NO is one of the main issues to decipher the signaling and metabolic functions of NO at each stage of the symbiotic interaction.

### NRs Regulate Energy State and Metabolism in Nitrogen-Fixing Nodules

The combined functioning of NR and mitochondrial ETC to produce NO has been associated with “Phytogb-NO” respiration and energy regeneration in hypoxic organs (Igamberdiev and Hill, 2009), and the role of NR in maintaining the ATP/ADP ratio has already been demonstrated in microoxic N_2_-fixing nodules of *M. truncatula* (Horchani et al., 2011). Our results (**Table 2**) show that, contrary to what is observed in 4 wpi-old nodules, the inhibition of NRs at the beginning of the symbiotic process (10 hpi and 4 dpi) does not cause a decrease in the ATP/ADP ratio indicating that, at these stages, energy regeneration is not linked to the functioning of Phytogb-NO respiration. These observations were expected since, contrary to mature nodules, roots are normoxic organs, and the cellular energy is supposed to be regenerated by the O_2_-dependent mitochondrial respiration. The role of mitochondrial ETC in NO production during the early days of the symbiotic process remains to be clarified.

The role of NR in energy, carbon, and nitrogen metabolism was investigated by using the NR inhibitor Tg. *In vivo* ^31^P-NMR experiments (**Figure 8A**) showed that hypoxia leads to a fall in ATP and to the acidification of cytoplasmic pH inside the nodules. These data are consistent with earlier observations with maize root tips (Roberts et al., 1984), soybean nodules (Pfeffer et al., 1992) and sycamore maple cells (Gout et al., 2001). In the presence of Tg, the fall in ATP and the cytoplasm acidification resulting from the inhibition of NR and their partial reversion by the addition of nitrite (**Figure 8B**) confirm that NR activity is fully involved in the energy metabolism of nodules *via* “Phytogb-NO” respiration, as already observed by Horchani et al. (2011). Logically, the inhibition of energy metabolism triggers the inhibition of the carbon and nitrogen metabolism in nodules, with an accumulation of sucrose, the subsequent decrease in the supply of carbon substrates to bacteroids (succinate, malate), and ultimately, the decrease in nitrogen reduction (ARA) and assimilation (asparagine) activities (**Figure 9**). It is interesting to note that treatment of nodules with Tg leads to an increase in alanine content (**Figure 9F**). In hypoxic/anoxic conditions, alanine accumulates in tissues following the inhibition of O_2_-dependent respiration and the induction of alanine aminotransferase (Limami et al., 2008, and references inside), and to date alanine is considered as one of the most prevalent and ubiquitous markers of hypoxia in plants (Gibbs and Greenway, 2003; Bailey-Serres and Voesenek, 2008; Limami et al., 2014). The accumulation of alanine, as well as the drop in the energy state (**Table 2**), is the demonstration that the inhibition of NR activity leads to the inhibition of the mitochondrial respiratory chain, the tricarboxylic acid cycle, and ultimately the carbon and nitrogen metabolism in the same way as the drop in O_2_ content in hypoxic tissue.

This conclusion is reinforced by the results obtained in the presence of nitrite. Interestingly, our study shows that over a short period of time, *i.e.* 4 h, nitrite stimulates ARA, while nitrate inhibits it (**Figure 9**). The inhibitory effect of nitrate on nitrogen fixation is widely recognized, and the question arose as to whether this effect was potentially linked to nitrite. Indeed, nitrite is known to be a potent inhibitor of nitrogenase *in vitro* (Wong, 1980; Trinchant and Rigaud, 1982). However, several studies have shown that nitrite is not responsible for the *in vivo* inhibition of nitrogenase activity induced by nitrate (Streeter, 1985a; Streeter, 1985b; Becana et al., 1989; Arrese-Igor et al., 1997; Arrese-Igor et al., 1998). The present study confirms this point and shows that over a short period of time, nitrite not only increases ARA, but even stimulates the energy, carbon, and nitrogen metabolism of nodules (**Figures 7****–****9**, **Table 2**). In a previous *in vivo* ^31^P-NMR study carried out on anoxic maize roots, Libourel et al. (2006) reported that the addition of nitrite makes it possible to limit the drop in cytoplasmic pH following anoxic treatment. Here, we show that 1) similarly to hypoxia, NR inhibition induces a drop in cytoplasmic pH, and 2) this drop in cytoplasmic pH may be reversed by the addition of nitrite (**Figure 8**), confirming the key role of NR and PNR, in the functioning of microoxic nodules.

Considered together, our data first confirm that NR is a source of nitrite, and indirectly of NO, during the establishment and functioning of N_2_-fixing symbiosis. Second, they support the hypothesis that NRs, in close collaboration with Phytogb1.1 (Berger et al., 2020), are strongly involved in the regulation of cellular energy and N_2_-fixing metabolism through the functioning of the Phytogb-NO respiration. Finally, they show that NR activity is needed for the N_2_-fixing symbiosis and constitutes a first attempt to explain its dual role in nodule functioning: 1) generating NO as a signal for gene regulation and metabolic adaptation, and 2) contributing to the energy supply under the hypoxic conditions prevailing inside the nodule.

## Data Availability Statement

All datasets generated for this study are included in the article/**Supplementary Material**.

## Author Contributions

ABe, ABo, DR, AP, and RB planned and designed the research. ABe, ABo, MM, MH, NH, SB, DR, and RB performed the experiments. ABe, ABo, DR, AP, and RB analyzed and interpreted the data. ABe, ABo, and RB wrote the manuscript.

## Conflict of Interest

The authors declare that the research was conducted in the absence of any commercial or financial relationships that could be construed as a potential conflict of interest.
