# Supplementary material for: Plant Nitrate Reductases Regulate Nitric Oxide Production and Nitrogen-Fixing Metabolism During the Medicago truncatula–Sinorhizobium meliloti Symbiosis
Source: Front Plant Sci. 2020 Sep 4;11:1313. doi: 10.3389/fpls.2020.01313 (PMC7500168; doi:10.3389/fpls.2020.01313)
Supplement: Supplementary file 1 [file Presentation_1.pptx]

## Slide 1
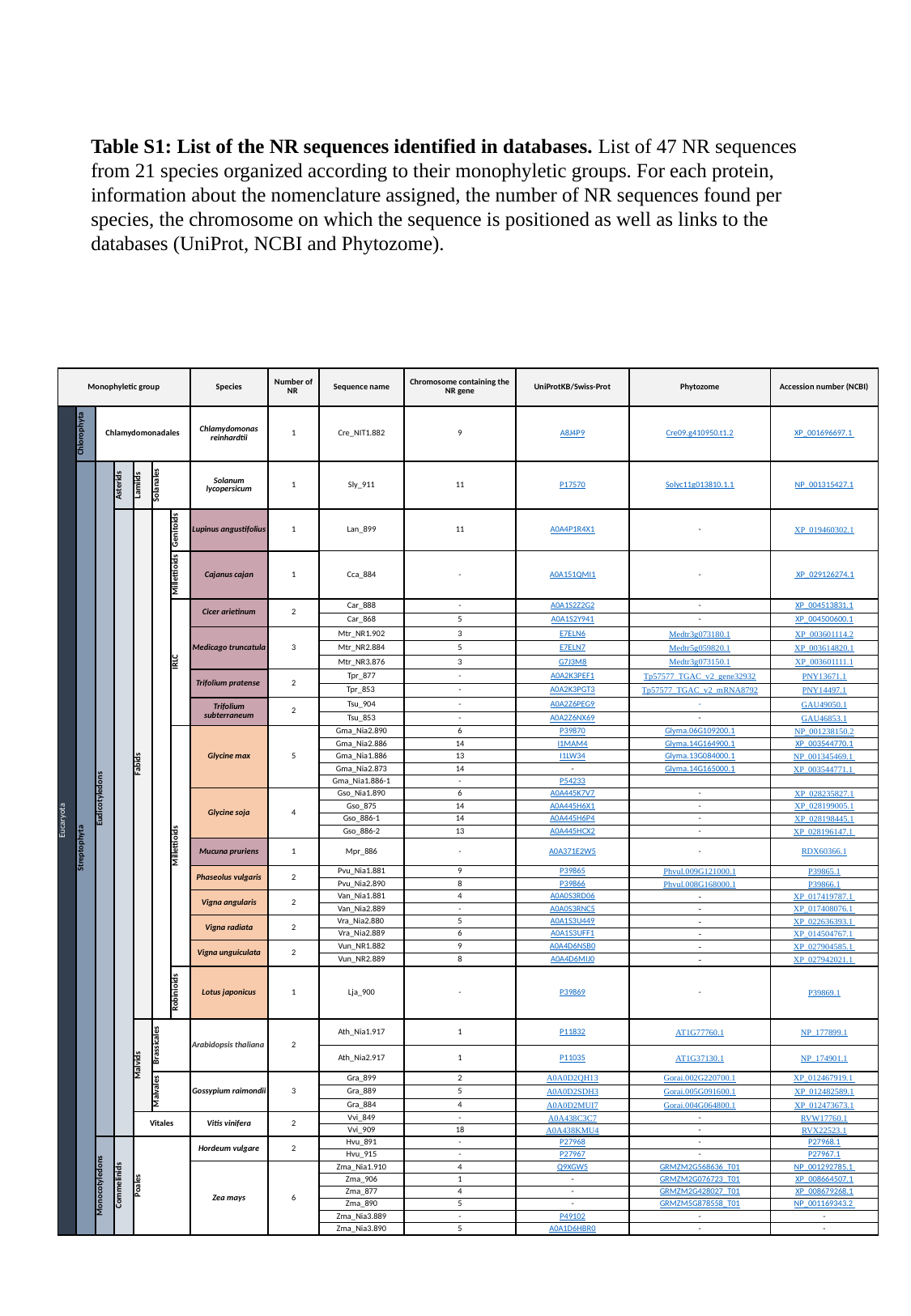

Table S1: List of the NR sequences identified in databases. List of 47 NR sequences from 21 species organized according to their monophyletic groups. For each protein, information about the nomenclature assigned, the number of NR sequences found per species, the chromosome on which the sequence is positioned as well as links to the databases (UniProt, NCBI and Phytozome).
| | | | | | | | | | | | | | | |
| --- | --- | --- | --- | --- | --- | --- | --- | --- | --- | --- | --- | --- | --- | --- |
| | Monophyletic group | | | | | | | Species | Number of NR | Sequence name | Chromosome containing the NR gene | UniProtKB/Swiss-Prot | Phytozome | Accession number (NCBI) |
| | | | | | | | | | | | | | | |
| | | | | | | | | | | | | | | |
| | Eucaryota | Chlorophyta | Chlamydomonadales | | | | | Chlamydomonas reinhardtii | 1 | Cre\_NIT1.882 | 9 | A8J4P9 | Cre09.g410950.t1.2 | XP\_001696697.1 |
| | | Streptophyta | Eudicotyledons | Asterids | Lamiids | Solanales | | Solanum lycopersicum | 1 | Sly\_911 | 11 | P17570 | Solyc11g013810.1.1 | NP\_001315427.1 |
| | | | | | Fabids | | Genitoids | Lupinus angustifolius | 1 | Lan\_899 | 11 | A0A4P1R4X1 | - | XP\_019460302.1 |
| | | | | | | | Millettioids | Cajanus cajan | 1 | Cca\_884 | - | A0A151QMI1 | - | XP\_029126274.1 |
| | | | | | | | IRLC | Cicer arietinum | 2 | Car\_888 | - | A0A1S2Z2G2 | - | XP\_004513831.1 |
| | | | | | | | | | | Car\_868 | 5 | A0A1S2Y941 | - | XP\_004500600.1 |
| | | | | | | | | Medicago truncatula | 3 | Mtr\_NR1.902 | 3 | E7ELN6 | Medtr3g073180.1 | XP\_003601114.2 |
| | | | | | | | | | | Mtr\_NR2.884 | 5 | E7ELN7 | Medtr5g059820.1 | XP\_003614820.1 |
| | | | | | | | | | | Mtr\_NR3.876 | 3 | G7J3M8 | Medtr3g073150.1 | XP\_003601111.1 |
| | | | | | | | | Trifolium pratense | 2 | Tpr\_877 | - | A0A2K3PEF1 | Tp57577\_TGAC\_v2\_gene32932 | PNY13671.1 |
| | | | | | | | | | | Tpr\_853 | - | A0A2K3PGT3 | Tp57577\_TGAC\_v2\_mRNA8792 | PNY14497.1 |
| | | | | | | | | Trifolium subterraneum | 2 | Tsu\_904 | - | A0A2Z6PEG9 | - | GAU49050.1 |
| | | | | | | | | | | Tsu\_853 | - | A0A2Z6NX69 | - | GAU46853.1 |
| | | | | | | | Millettioids | Glycine max | 5 | Gma\_Nia2.890 | 6 | P39870 | Glyma.06G109200.1 | NP\_001238150.2 |
| | | | | | | | | | | Gma\_Nia2.886 | 14 | I1MAM4 | Glyma.14G164900.1 | XP\_003544770.1 |
| | | | | | | | | | | Gma\_Nia1.886 | 13 | I1LW34 | Glyma.13G084000.1 | NP\_001345469.1 |
| | | | | | | | | | | Gma\_Nia2.873 | 14 | - | Glyma.14G165000.1 | XP\_003544771.1 |
| | | | | | | | | | | Gma\_Nia1.886-1 | - | P54233 | | |
| | | | | | | | | Glycine soja | 4 | Gso\_Nia1.890 | 6 | A0A445K7V7 | - | XP\_028235827.1 |
| | | | | | | | | | | Gso\_875 | 14 | A0A445H6X1 | - | XP\_028199005.1 |
| | | | | | | | | | | Gso\_886-1 | 14 | A0A445H6P4 | - | XP\_028198445.1 |
| | | | | | | | | | | Gso\_886-2 | 13 | A0A445HCX2 | - | XP\_028196147.1 |
| | | | | | | | | Mucuna pruriens | 1 | Mpr\_886 | - | A0A371E2W5 | - | RDX60366.1 |
| | | | | | | | | Phaseolus vulgaris | 2 | Pvu\_Nia1.881 | 9 | P39865 | Phvul.009G121000.1 | P39865.1 |
| | | | | | | | | | | Pvu\_Nia2.890 | 8 | P39866 | Phvul.008G168000.1 | P39866.1 |
| | | | | | | | | Vigna angularis | 2 | Van\_Nia1.881 | 4 | A0A0S3RD06 | - | XP\_017419787.1 |
| | | | | | | | | | | Van\_Nia2.889 | - | A0A0S3RNC5 | - | XP\_017408076.1 |
| | | | | | | | | Vigna radiata | 2 | Vra\_Nia2.880 | 5 | A0A1S3U449 | - | XP\_022636393.1 |
| | | | | | | | | | | Vra\_Nia2.889 | 6 | A0A1S3UFF1 | - | XP\_014504767.1 |
| | | | | | | | | Vigna unguiculata | 2 | Vun\_NR1.882 | 9 | A0A4D6NSB0 | - | XP\_027904585.1 |
| | | | | | | | | | | Vun\_NR2.889 | 8 | A0A4D6MIJ0 | - | XP\_027942021.1 |
| | | | | | | | Robinioids | Lotus japonicus | 1 | Lja\_900 | - | P39869 | - | P39869.1 |
| | | | | | Malvids | Brassicales | | Arabidopsis thaliana | 2 | Ath\_Nia1.917 | 1 | P11832 | AT1G77760.1 | NP\_177899.1 |
| | | | | | | | | | | Ath\_Nia2.917 | 1 | P11035 | AT1G37130.1 | NP\_174901.1 |
| | | | | | | Malvales | | Gossypium raimondii | 3 | Gra\_899 | 2 | A0A0D2QH13 | Gorai.002G220700.1 | XP\_012467919.1 |
| | | | | | | | | | | Gra\_889 | 5 | A0A0D2SDH3 | Gorai.005G091600.1 | XP\_012482589.1 |
| | | | | | | | | | | Gra\_884 | 4 | A0A0D2MUI7 | Gorai.004G064800.1 | XP\_012473673.1 |
| | | | | | Vitales | | | Vitis vinifera | 2 | Vvi\_849 | - | A0A438C3C7 | - | RVW17760.1 |
| | | | | | | | | | | Vvi\_909 | 18 | A0A438KMU4 | - | RVX22523.1 |
| | | | Monocotyledons | Commelinids | Poales | | | Hordeum vulgare | 2 | Hvu\_891 | - | P27968 | - | P27968.1 |
| | | | | | | | | | | Hvu\_915 | - | P27967 | - | P27967.1 |
| | | | | | | | | Zea mays | 6 | Zma\_Nia1.910 | 4 | Q9XGW5 | GRMZM2G568636\_T01 | NP\_001292785.1 |
| | | | | | | | | | | Zma\_906 | 1 | - | GRMZM2G076723\_T01 | XP\_008664507.1 |
| | | | | | | | | | | Zma\_877 | 4 | - | GRMZM2G428027\_T01 | XP\_008679268.1 |
| | | | | | | | | | | Zma\_890 | 5 | - | GRMZM5G878558\_T01 | NP\_001169343.2 |
| | | | | | | | | | | Zma\_Nia3.889 | - | P49102 | - | - |
| | | | | | | | | | | Zma\_Nia3.890 | 5 | A0A1D6HBR0 | - | - |
| | | | | | | | | | | | | | | |

## Slide 2
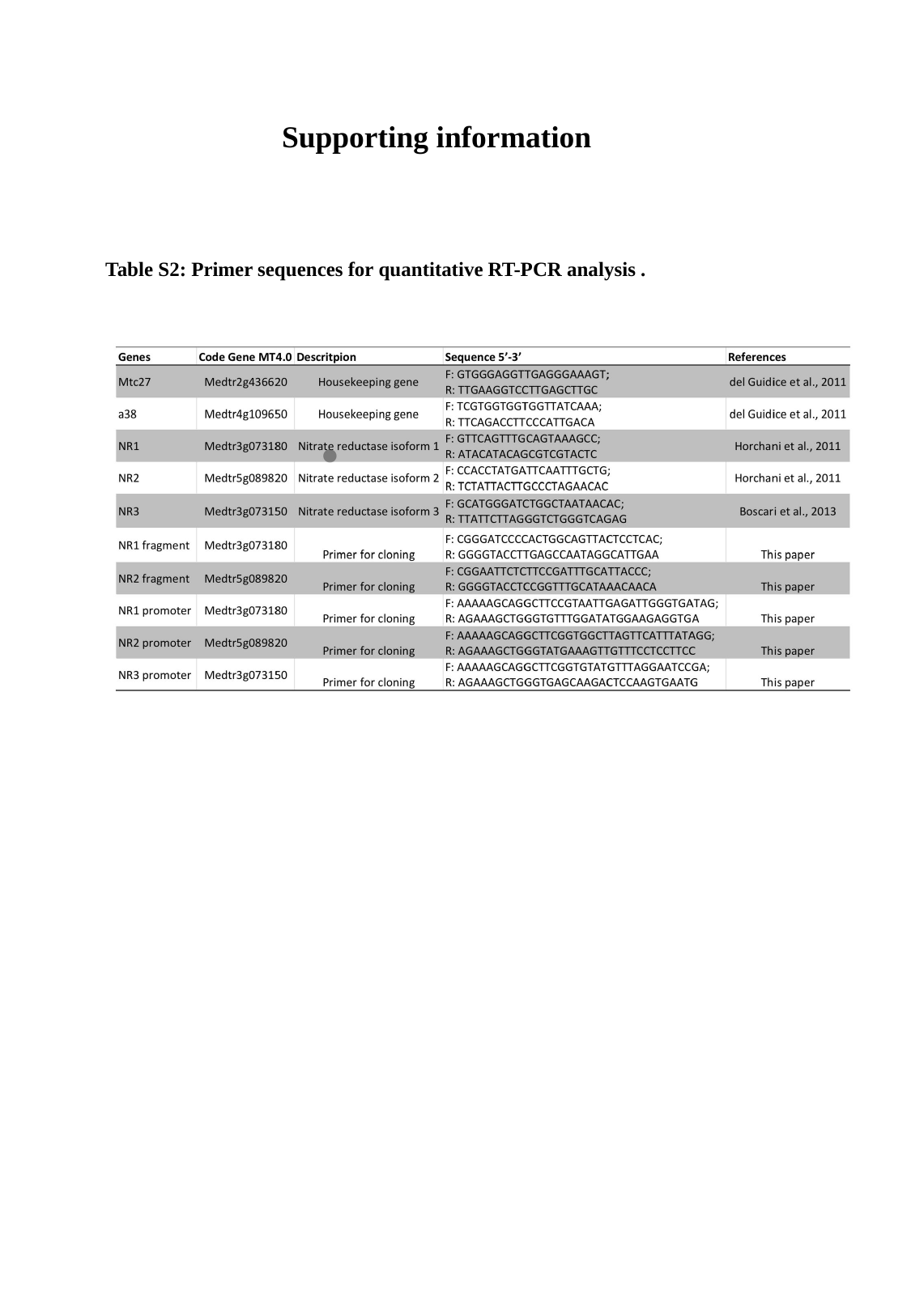

Supporting information
Table S2: Primer sequences for quantitative RT-PCR analysis .

## Slide 3
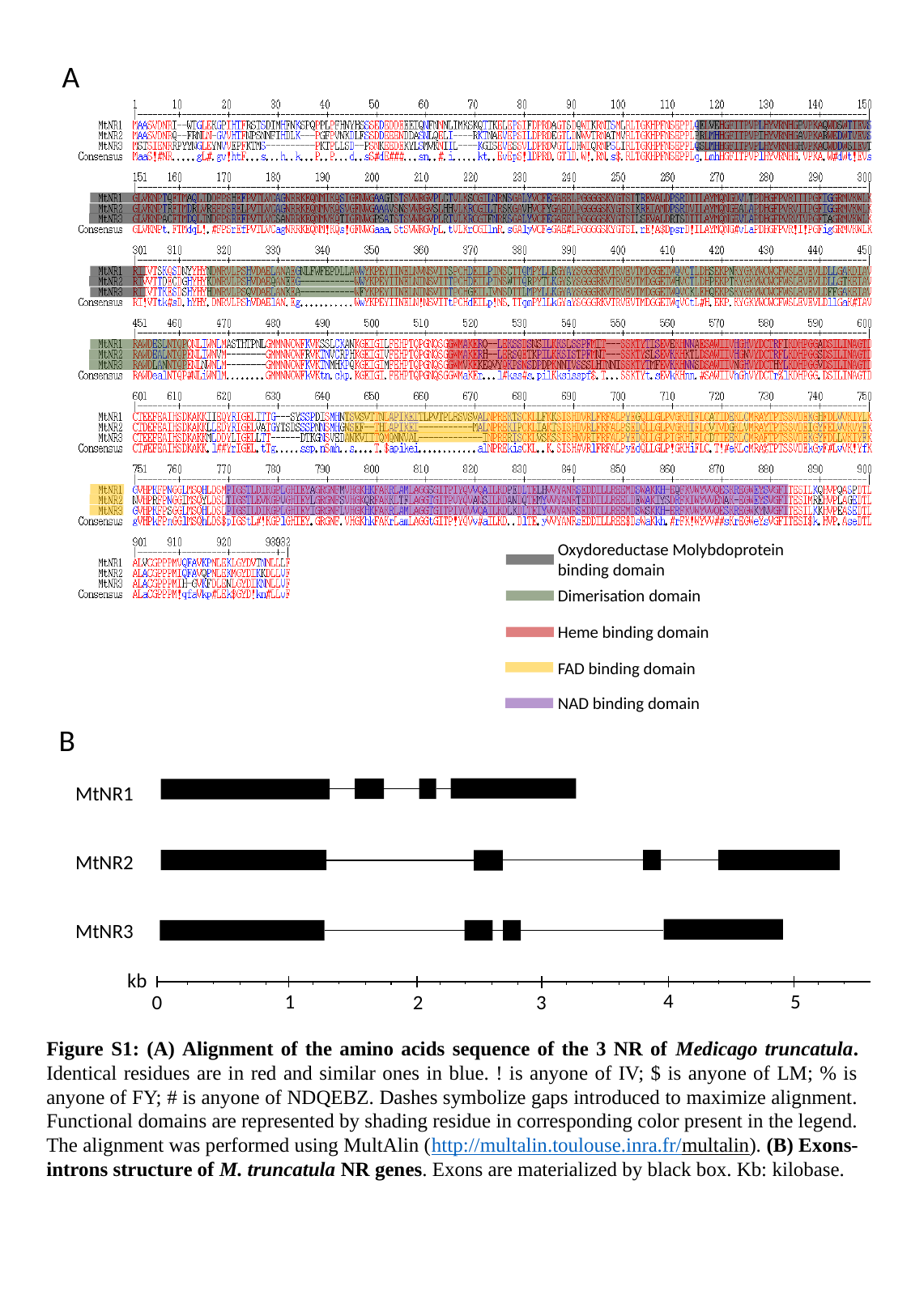

A
Oxydoreductase Molybdoprotein binding domain
Dimerisation domain
Heme binding domain
FAD binding domain
NAD binding domain
B
MtNR1
MtNR2
MtNR3
kb
4
5
1
3
2
0
Figure S1: (A) Alignment of the amino acids sequence of the 3 NR of Medicago truncatula. Identical residues are in red and similar ones in blue. ! is anyone of IV; $ is anyone of LM; % is anyone of FY; # is anyone of NDQEBZ. Dashes symbolize gaps introduced to maximize alignment. Functional domains are represented by shading residue in corresponding color present in the legend. The alignment was performed using MultAlin (http://multalin.toulouse.inra.fr/multalin). (B) Exons-introns structure of M. truncatula NR genes. Exons are materialized by black box. Kb: kilobase.

## Slide 4
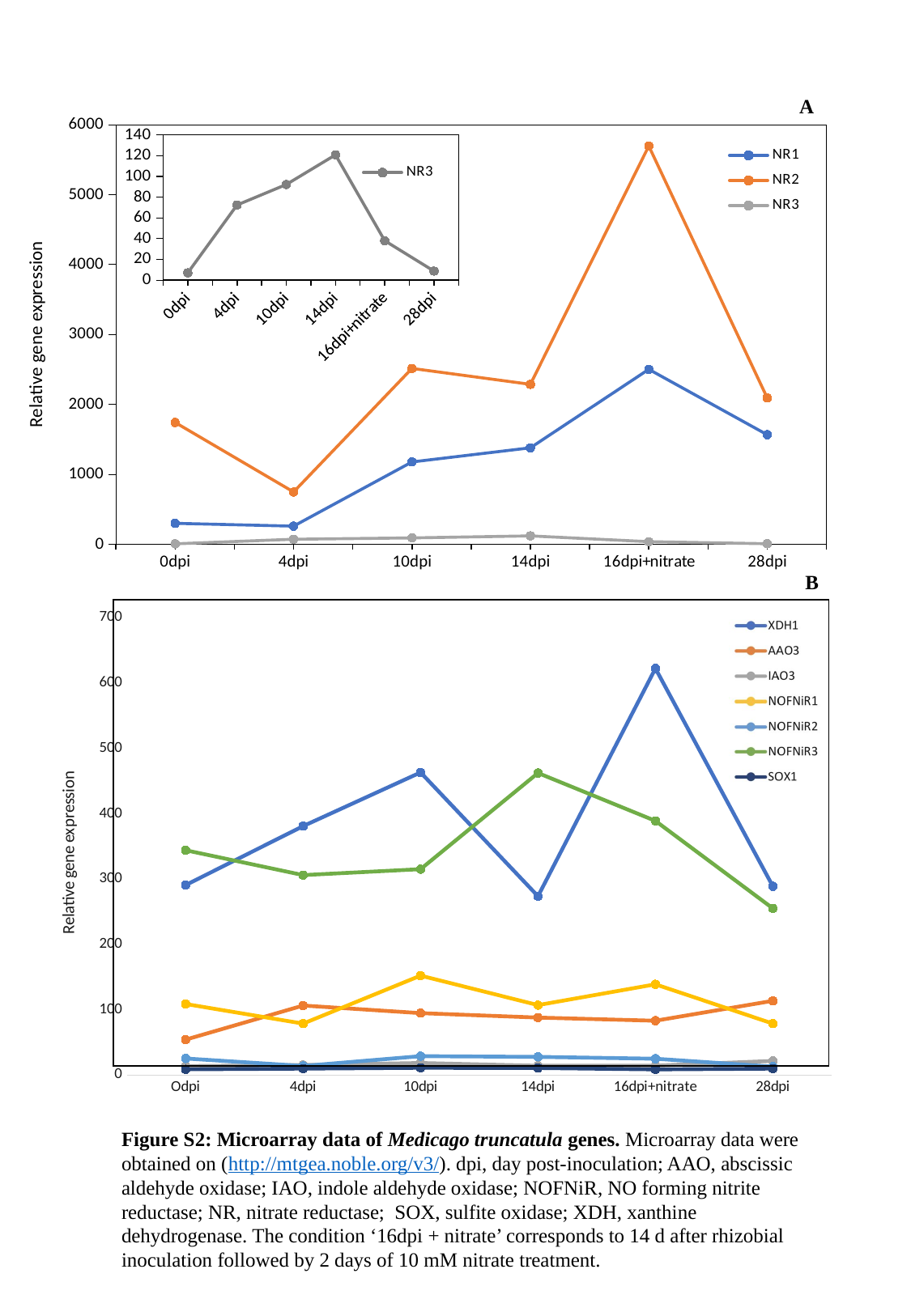

A
### Chart
| Category | NR1 | NR2 | NR3 |
|---|---|---|---|
| 0dpi | 300.36 | 1741.76 | 7.02 |
| 4dpi | 259.71 | 748.22 | 72.47 |
| 10dpi | 1177.41 | 2514.6 | 92.24 |
| 14dpi | 1380.12 | 2287.11 | 120.83 |
| 16dpi+nitrate | 2501.88 | 5695.19 | 38.02 |
| 28dpi | 1567.16 | 2094.15 | 8.69 |
### Chart
| Category | NR3 |
|---|---|
| 0dpi | 7.02 |
| 4dpi | 72.47 |
| 10dpi | 92.24 |
| 14dpi | 120.83 |
| 16dpi+nitrate | 38.02 |
| 28dpi | 8.69 |Relative gene expression
B
### Chart
| Category | XDH1 | AAO3 | IAO3 | NOFNiR1 | NOFNiR2 | NOFNiR3 | SOX1 |
|---|---|---|---|---|---|---|---|
| Odpi | 290.71 | 54.14 | 13.65 | 108.65 | 25.29 | 343.97 | 8.72 |
| 4dpi | 380.96 | 106.32 | 15.22 | 78.69 | 13.85 | 305.9 | 9.76 |
| 10dpi | 463.06 | 94.66 | 18.43 | 152.1 | 28.88 | 315.0 | 10.99 |
| 14dpi | 273.56 | 87.89 | 14.13 | 106.94 | 27.79 | 462.22 | 10.71 |
| 16dpi+nitrate | 622.24 | 83.08 | 14.36 | 138.79 | 24.9 | 388.94 | 8.62 |
| 28dpi | 288.67 | 113.56 | 21.52 | 78.68 | 12.84 | 254.91 | 9.78 |
Figure S2: Microarray data of Medicago truncatula genes. Microarray data were obtained on (http://mtgea.noble.org/v3/). dpi, day post-inoculation; AAO, abscissic aldehyde oxidase; IAO, indole aldehyde oxidase; NOFNiR, NO forming nitrite reductase; NR, nitrate reductase; SOX, sulfite oxidase; XDH, xanthine dehydrogenase. The condition ‘16dpi + nitrate’ corresponds to 14 d after rhizobial inoculation followed by 2 days of 10 mM nitrate treatment.

## Slide 5
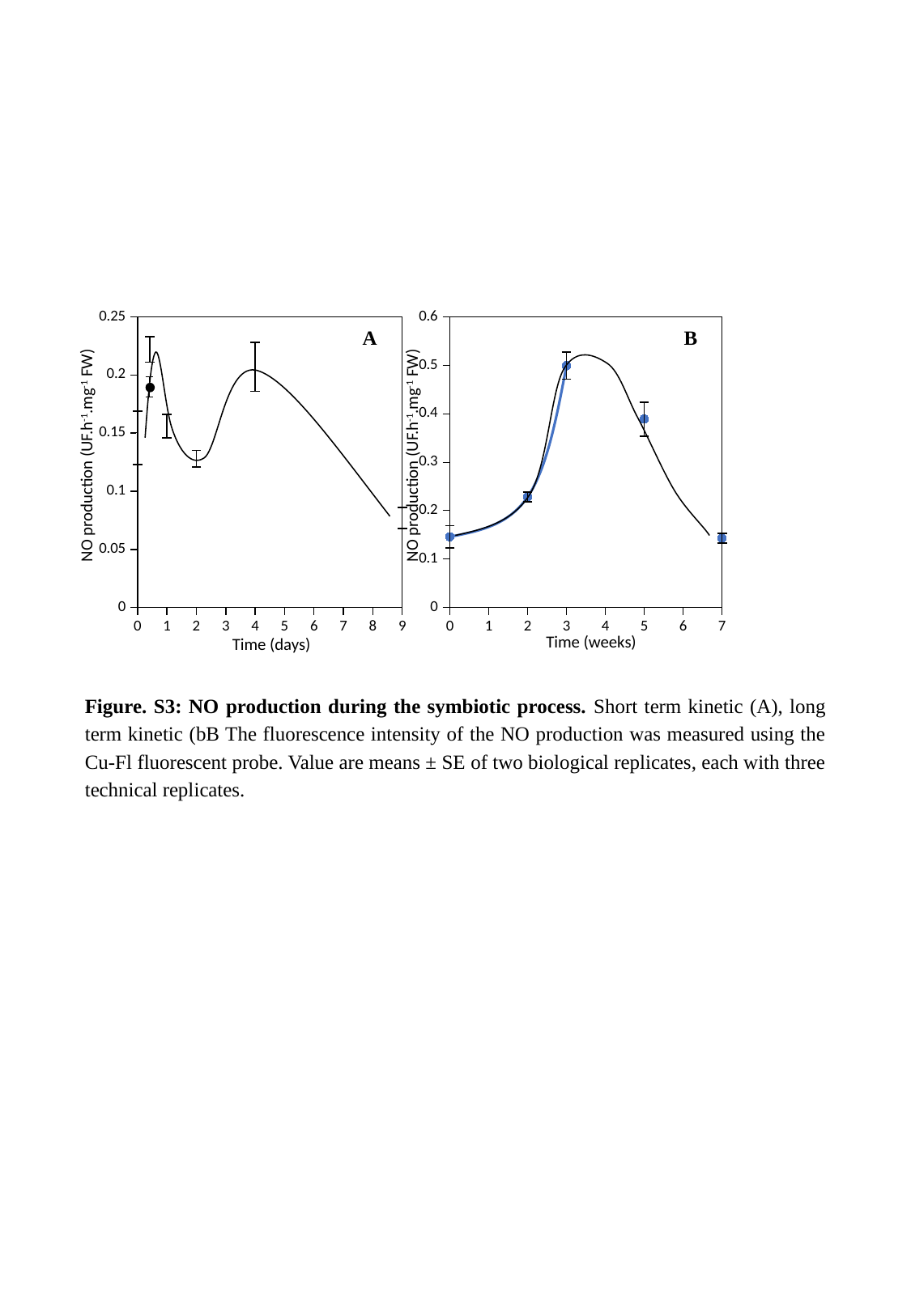

### Chart
| Category | |
|---|---|
### Chart
| Category | |
|---|---|A
B
NO production (UF.h-1.mg-1 FW)
NO production (UF.h-1.mg-1 FW)
Time (weeks)
Time (days)
Figure. S3: NO production during the symbiotic process. Short term kinetic (A), long term kinetic (bB The fluorescence intensity of the NO production was measured using the Cu-Fl fluorescent probe. Value are means ± SE of two biological replicates, each with three technical replicates.
